# Duplication of a Single *myhz1.1* Gene Facilitated the Ability of Goldfish (*Carassius auratus*) to Alter Fast Muscle Contractile Properties With Seasonal Temperature Change

**DOI:** 10.3389/fphys.2018.01724

**Published:** 2018-12-04

**Authors:** Daniel Garcia de la serrana, Kristin Wreggelsworth, Ian A. Johnston

**Affiliations:** ^1^School of Biology, Scottish Oceans Institute, University of St. Andrews, St Andrews, United Kingdom; ^2^Serra Húnter Fellow, Cell Biology Physiology and Immunology Department, School of Biology, Universitat de Barcelona, Barcelona, Spain

**Keywords:** skeletal muscle, teleost, myosin heavy chain, temperature, gene duplication

## Abstract

Seasonal temperature changes markedly effect the swimming performance of some cyprinid fish acutely tested at different temperatures, involving a restructuring of skeletal muscle phenotype including changes in contractile properties and myosin heavy chain expression. We analyzed the transcriptome of fast myotomal muscle from goldfish (*Carassius auratus* L.) acclimated to either 8 or 25°C for 4 weeks (12 h light: 12 h dark) and identified 10 myosin heavy chains (*myh*) and 13 myosin light chain (*myl*) transcripts. Goldfish orthologs were classified based on zebrafish nomenclature as *myhz1.1α*, *myhz1.1β*, *myhz1.1γ*, *myha*, *myhb*, *embryo_myh1, myh9b, smyh2*, *symh3*, and *myh11* (myosin heavy chains) *and myl1a*, *myl1b*, *myl2*, *myl9a*, *myl9b*, *myl3*, *myl13*, *myl6, myl12.1a, myl12.1b, myl12.2a, myl12.2b*, and *myl10* (myosin light chains). The most abundantly expressed transcripts *myhz1.1α*, *myhz1.1β*, *myhz1.1γ*, *myha*, *myl1a*, *myl1b*, *myl2*, and *myl3*) were further investigated in fast skeletal muscle of goldfish acclimated to either 4, 8, 15, or 30°C for 12 weeks (12 h light:12 h dark). Total copy number for the myosin heavy chains showed a distinct optimum at 15°C (*P* < 0.01). Together m*yhz1.1α* and *myhz1.1β* comprised 90 to 97% of myhc transcripts below 15°C, but only 62% at 30°C. Whereas *myhz1.1α* and *myhz1.1β* were equally abundant at 4 and 8°C, *myhz1.1β* transcripts were 17 and 12 times higher than *myhz1.1α* at 15 and 30°C, respectively, (*P* < 0.01). M*yhz1.1γ* expression was at least nine-fold higher at 30°C than at cooler temperatures (*P* < 0.01). In contrast, the expression of *myha* and myosin light chains showed no consistent pattern with acclimation temperature. A phylogenetic analysis indicated that the previously reported ability of goldfish and common carp to alter contractile properties and myofibrillar ATPase activity with temperature acclimation was related to the duplication of a single *myhz1.1* fast muscle myosin heavy chain found in basal cyprinids such as the zebrafish (*Danio rerio*).

## Introduction

The temperature of freshwater lakes and all but the smallest ponds changes more slowly seasonally than the air temperature providing a stable cue that allows phenotypic plasticity to act as a compensatory mechanism to adjust physiology and behavior to the prevailing environmental conditions (Johnston and Temple, 2002; Seebacher et al., 2012; Jeffries et al., 2016). These phenomena have been particularly studied in skeletal muscle because of the relative ease of interpreting changes at the molecular and sarcomere levels in terms of altered contractile performance and swimming behavior (reviewed in Johnston, 2006). Such phenotypic plasticity is commonly observed in cyprinid fish including goldfish, crucian carp, common carp, and grass carp that experience relatively large difference between summer and winter temperatures of 25°C or more (Fry and Hart, 1948; Johnston et al., 1975; Johnston and Maitland, 1980; Imai et al., 1996; Tao et al., 2005). In contrast, species of the same family inhabiting habitats with a seasonal range of 15°C or less including the model zebrafish (*Danio* *rerio*) have temperature-performance profiles that are unaltered by several months acclimation to the average maximum or minimum seasonal temperature (McClelland et al., 2006).

One of the earliest papers documenting temperature acclimation responses in fish measured the maximum speed that goldfish (*Carassius auratus*) could maintain in a rotating swimming chamber at various test temperatures for a fixed duration of 2 min (Fry and Hart, 1948). They found that temperature-maximum performance curves were markedly different in fish acclimated for several weeks to different test temperatures. Cold-acclimation improved performance at low temperatures but resulted in reduced swimming performance at high test temperatures and vice versa (Fry and Hart, 1948). Cold-acclimation was subsequently shown to result in a more aerobic phenotype in both fast and slow muscle fibers (Johnston and Maitland, 1980; Sidell, 1980) and higher myofibrillar ATPase activity at low test temperatures (Johnston et al., 1975). Contractile phenotype was also profoundly modified by temperature acclimation in the common carp (*Cyprinus carpio*) including the amount of sarcoplasmic reticulum, twitch contraction times (Fleming et al., 1990) and the force-velocity relationship (Johnston et al., 1985). At the molecular level temperature acclimation was found to modify the predominant isoform of myosin heavy chain expressed in fast muscle in both common carp (Imai et al., 1996) and grass carp (*Ctenopharyngodon idella*) acclimated to either 10 or 30°C for 5–8 weeks (Tao et al., 2009). In both species, three myosin heavy chains were expressed in an acclimation-temperature dependent manner and named according to their predominant expression temperature as 10°C-type, intermediate-type and 30°C-type (Imai et al., 1996; Tao et al., 2005, 2009). In contrast, temperature acclimation did not alter the sub-unit composition of myosin light chains (Hwang et al., 1990).

In the present study, the transcriptome of fast myotomal muscle was characterized in goldfish acclimated to either 8 or 30°C for 4 weeks to identify the most abundant myosin heavy chain (*myh*) and myosin light chains (*myl*) expressed. Phylogenetic analysis was used to provide an insight into the evolution of myosin heavy chains in the Cypriniformes lineage that have expression patterns which are seasonally regulated. Myosin genes in the goldfish were named according to the nomenclature used for the zebrafish (*Danio rerio*), a more basal cyprinid (Wang et al., 2007) with a more restricted temperature range (15–30°C) that is unable to modify swimming performance following temperature acclimation (McClelland et al., 2006). To further investigate the transcriptional regulation of myosin, the copy number of the predominantly expressed *myh* and *myl* chain transcripts was then quantified in a second experiment for goldfish acclimated to either 4, 8, 15, or 30°C for a period of 12 weeks.

## Materials and Methods

### Fish and Experimental Conditions

All experimental procedures were approved by the Animal Welfare and Ethics Committee (AWEC) of the University of St Andrews. Goldfish (*Carassius auratus* L.) were obtained from accredited suppliers and maintained in 200 L tanks of temperature-controlled freshwater with 96% recirculation (12 h light: 12 h dark) in the Scottish Oceans Institute aquarium facilities. Fish were hand fed with a commercial diet at maintenance level. In a preliminary study of the fast muscle transcriptome goldfish of 20–30 g body mass were acclimated to either 8 or 25°C for 4 weeks. The main experiment comprised four groups of 30 fish of similar body size that were maintained in duplicated tanks and acclimated to 15°C for 2 weeks. The temperature of each replicate tank was adjusted at the rate of 1°C day^-1^ to final acclimation temperatures of 4, 8, 15, or 30°C (12 h light: 12 h dark). Fish were maintained at these temperatures for 12 weeks before being humanely sacrificed using a schedule-1 United Kingdom Home Office approved protocol involving a sharp blow in the head followed by sectioning of the spinal cord. The body size of the different temperature acclimation groups was not significantly different at the end of the experiment (*P* > 0.5; Supplementary File S1).

### Tissue Sampling

All tissue samples were taken from deep layers of the dorsal myotomal muscle at the level of the dorsal fin which consists of pure fast muscle fibers. Around 1 g of fast muscle was dissected per fish for RNA extraction, snap frozen in liquid nitrogen and stored at -80°C until further analysis.

### RNA Extraction

Between 20 and 40 mg of pure fast skeletal muscle was used for total RNA extraction using TriSure reagent (Bioline, London, United Kingdom) following the manufacturer’s recommendations. Briefly, tissue was homogenized in 1 ml TriSure using MatrixD (MP Biomedicals), vigorously mixed with 200 μl of chloroform and centrifuged. The upper liquid phase was recovered, mixed with 500 μl of 2-propanol and centrifuged again to precipitate the RNA. The resulting pellet was washed three times with 1 ml of cold 70% ethanol, dried and diluted in 50 μl of RNAse free water. Total RNA concentration, 260/280 and 260/230 ratios were estimated using a Nanodrop 1000 spectrophotometer (Thermo Fisher Scientific, United Kingdom) and integrity was evaluated in a 1.5% (m/v) ethidium bromide agarose gel. All samples used had non-degraded RNA with 260/280 and 260/230 ratios over 2.

### Fast Muscle Transcriptome

Equal amounts of total RNA from 5 goldfish per acclimation temperature (8 and 25°C for 4 weeks) were pooled. The two pools were pair-end sequenced on one lane of an Illumina Miseq using v3 chemistry by Eurofins Genomics (ENA Accession Number: PRJEB17982). Low quality reads and adaptors were removed and remaining reads assembled using Eurofins in-house scripts combining Velvet (Zerbino and Birney, 2008) and Oases (Schulz et al., 2012) assembler software. In order to reduce redundancy from the initial assembly, contigs were re-clustered based on sequence identity using cd-hit-EST (Li and Godzik, 2006) with an identity threshold of 99%. Contigs obtained after the clustering were blasted (BLASTx) against the NCBI non-redundant database. Gene Ontology (GO) annotation was performed using BLAST2GO software (Götz et al., 2008) with an *e*-value threshold of 10^-3^.

Sarcomere protein components were identified in the *de novo* transcriptome using two complementary strategies: (1) amino acid sequences for myosin heavy chain and myosin light chain orthologs for zebrafish were retrieved from the available genome^1^ and BLAST (tBLASTn) against the *de novo* goldfish transcriptome (2) in parallel all nucleotide sequences of myosin sub-units available for common carp (*Cyprinus carpio*) were also retrieved from NCBI public database^2^ and genome assembly^3^ and BLAST (BLASTn) against the goldfish transcriptome. Positive hits from both approaches were retrieved and re-BLAST against the NCBI non-redundant (nr) database to confirm their identity.

Orthologs for *myosin heavy chain* and *myosin light chain genes* were retrieved from the Ensembl database^1^ for *Oreochromis niloticus*, *Gasterosteus aculeatus*, *Danio rerio*, *Astyanax mexicanus*, *Oryzias latipes*, *Tetraodon nigroviridis*, *Homo sapiens*, and *Mus musculus* (all accession number are provided in Supplementary File S2). Nucleotide sequences were aligned using the GUIDANCE2 online server (Sela et al., 2015) with PRANK as the multi-sequence alignment algorithm. Columns below the 0.93 GUIDANCE2 quality alignment score were removed and remaining aligned sequences used for phylogenetic reconstruction. The best evolutionary model for each alignment was estimated using MEGA7 (Kumar et al., 2016). Bayesian MCMC phylogenetic trees, following a Yule speciation process model and UPGMA starting tree, were generated using BEAST v1.7.5 software with 10,000,000 random seeds (Drummond et al., 2012). Final Bayesian trees were generated using TreeAnnotator v1.7.5 with a burning value of 1,000. All trees were visualized using FigTree v1.3.1.

### Validation of Paralogous Sequences

Potential gene paralogs identified in the *de novo* transcriptome were experimentally validated. In brief, primers targeting amplicons between 500–1000 bp were design in the most divergent region of the potential paralogs in order to favor the amplification of an individual gene (Supplementary File S3). Paralogs were amplified using standard PCR procedures using the following protocol: 95°C 5 min, 25 × (95°C 30 s, 60°C 30 s, and 72°C 1.5 min) followed by a final extension step of 7 min at 72°C. PCR reactions were resolved in a 2% (m/v) ethidium bromide gel and bands of expected size were cut-out and stored at -20°C prior to extraction. PCR amplicons were extracted, from agarose gels using the QIAquick extraction kit (QIAGEN) and ligated to a TOPO^®^ vector (Thermofisher, Paisley, United Kingdom) containing a resistance to ampicillin, transformed into chemically competent *E. coli* by thermal shock and incubated 1 h at 37°C in 200 μl of SOC medium following the manufacturer’s recommendations (Thermofisher). A total of 100 μl of the transformed *E. coli* were plated in LB-agarose plates containing 75 ng/ml ampicillin (Thermofisher) and incubated for 16 h at 37°C. A total of 8 clones per product were growth in LB-broth medium at 37°C for 16 h and 1 μl of bacteria was amplified by PCR using T7/T3 primers to confirm the presence of the right-sized insert in all selected clones. Miniprep extractions were performed for those clones with the correct insert size and plasmid concentration estimated by Nanodrop spectrometry. All clones were Sanger sequenced by the University of Dundee Sequencing Service.

### cDNA Synthesis and Quantitative PCR

1 μg of total RNA per individual was reverse transcribed using Quantitect cDNA synthesis kit (Qiagen) including a gDNA wipe-out step to remove any remnants of genomic DNA. Six microliters per sample were mixed with 7.5 μl of SensiFast SYBR Lo-ROX 2x master mix (Bioline, London, United Kingdom) containing 400 nmol^-1^ sense/antisense primers. Reactions were performed in duplicate using a Mx3005P Thermocycler (Agilent, Berkshire, United Kingdom), with one cycle of 2 min at 95°C and 40 cycles of 5 s at 95°C and 20 s at 65°C, followed by a dissociation curve analysis, which resulted in a single peak for all qPCR reactions analyzed.

Primers were designed using Primer 3 (Untergasser et al., 2012) to anneal at 60°C and amplify products between 100–250 bp (Supplementary File S3). NetPrimer (PremierBiosoft) was used to test for primer hairpins, self-dimmers, and cross-dimmers. In the case of confirmed paralogs pairs, primers were designed in the most divergent fragments targeting differences in the 3′ region of the sequence as previously described (Garcia de la serrana et al., 2012; Garcia de la serrana and Johnston, 2013). Gene efficiency was estimated by a dilution series of a pooled sample. Housekeeping genes *beta actin* (*ß-actin*), *elongation factor 1 alpha* (*ef1a*), and *ribosomal protein 27* (*rpl27*) were tested for stability using BestKeeper (Pfaffl et al., 2004) and *rpl27* was selected for gene expression normalization. All qPCR reactions were resolved in a 2% (m/v) ethidium-bromide agarose gel and specific bands were purified from the gel using a QIAgel extraction kit (QIAGEN) and sequenced at the University of Dundee Sequencing Service. For absolute quantification of transcript abundance, qPCR fragments were cloned in plasmids and purified as described above. A calibration curve with 10, 1, 0.1, 0.01, and 0.001 nanograms of plasmid for each insert were constructed and the qPCR protocol described above followed. The calibrations curves were used to translate Ct from each of the genes analyzed to number of copies per μg of cDNA. Transcript abundance of myosin heavy chains and myosin light chains was normalized against by dividing the gene total number of transcripts by the housekeeping (*rpl27*) total number of transcripts.

### Statistical Analysis

All statistical analyses were conducted using R-Studio v.1.1.419. Shapiro-Wilk was used to test normality of the gene expression data. Differences in gene expression were analyzed using normalized expression values by ANOVA test with *temperature* as factor followed by a Tukey *post hoc* test. *P*-values were corrected by multiple comparison applying a false discovery rate (FDR) correction. Differences were considered significant when FDR < 0.05. All graphs were produced using the ggplot2 R-build package (Wickham, 2016).

## Results

### Fast Skeletal Muscle Transcriptome

A total of 7.5 and 10.0 million paired-end reads (190 bp) were obtained by Illumina sequencing of cDNA libraries prepared from the fast myotomal muscle of goldfish acclimated to either 25 or 8°C, respectively. Following assembly, the number or transcripts obtained was 66,752 (25°C library) and 93,586 (8°C library) (Table 1). Transcripts longer than 1,000 bp comprised 23% of the 25°C library and 40% of the 8°C library (Table 1). The transcripts coding for subunits of myosin were exhaustively characterized using the combined transcriptome.

**Table 1:** Summary statistics for goldfish transcriptomes.

Parameter	Temperature	
	25°C	8°C
Paired reads	7952911	9408663
Average read length (bp)	191	191
Assembled reads	6166316	7442177
All transcripts	66752	93586
Average transcript length (bp)	821	1182
Long Transcripts (>1000 bp)	15666	37306

*bp = base pairs.*

#### Myosin Heavy Chains (*myh*)

**FIGURE 1:**
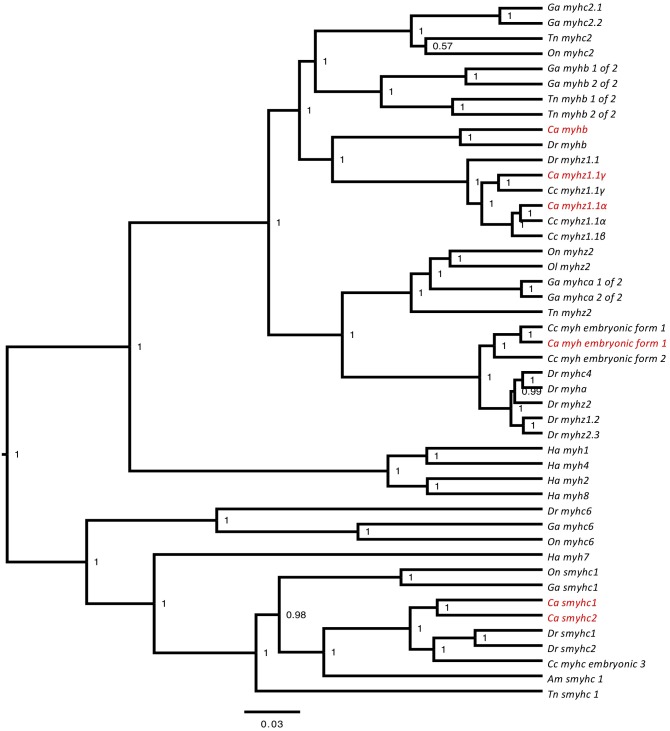
Phylogenetic tree constructed for myosin heavy chain genes. Bayesian phylogenetic relationships for the myosin heavy chain orthologs. Phylogenetic tree node numbers represent posterior values. To facilitate interpretation, goldfish orthologs (*Carassius auratus, Ca*) are highlighted in red. Myosin heavy chain orthologs for zebrafish (*Danio rerio, Dr*), stickleback (*Gasterosteus aculeatus, Ga*), common carp (*Cyprinus carpio, Cc*), green spotted pufferfish (*Tetraodon nigroviridis, Tn*), tilapia (*Oreochromis niloticus, On*), medaka (*Oryzias latipes, Ol*), and human (*Homo sapiens, Ha*) were also included.

To identify *myh* transcripts the transcriptome was successively BLAST against the zebrafish genome (vz9) and myhc transcripts from the common carp (*Cyprinus carpio*) another cyprinid fish which differentially regulates *myh* expression with temperature acclimation (Imai et al., 1996). A total of 10 different myosin heavy chain transcripts were identified of which 8 were of sufficient length for further validation by phylogenetic analysis (Supplementary File S4). The goldfish myosin heavy chain orthologs were named according to the zebrafish nomenclature as *myhz1.1α*, *myhz1.1β*, *myhz1.1γ*, *myha*, *myhb*, *smyh2*, *smyh3*, *embryo_myh1*, *myh10*, and *myh11* (Figure 1). In the case of embryo_myh1 gene, blast results indicated a equal similarity to zebrafish *myhz2, myhz1.3*, and *myhc4* (data not show) but since the phylogenetic analysis did not revolve its identity (Figure 1) the previous cyprinid nomenclature was maintained. The *myhz1.1α*, *myhz1.1β*, and *myhz1.1γ* transcripts were orthologous to the major fast muscle myosin heavy chain isoforms expressed in the common carp at acclimation temperatures of 10, 15, and 30°C, respectively, (Imai et al., 1996) and to a single ortholog of *myhz1.1* in zebrafish. *Myha* and *myhb* are also characterized as fast muscle genes in the zebrafish genome assembly (Nord et al., 2014).

#### Myosin Light Chains (*myl*)

**FIGURE 2:**
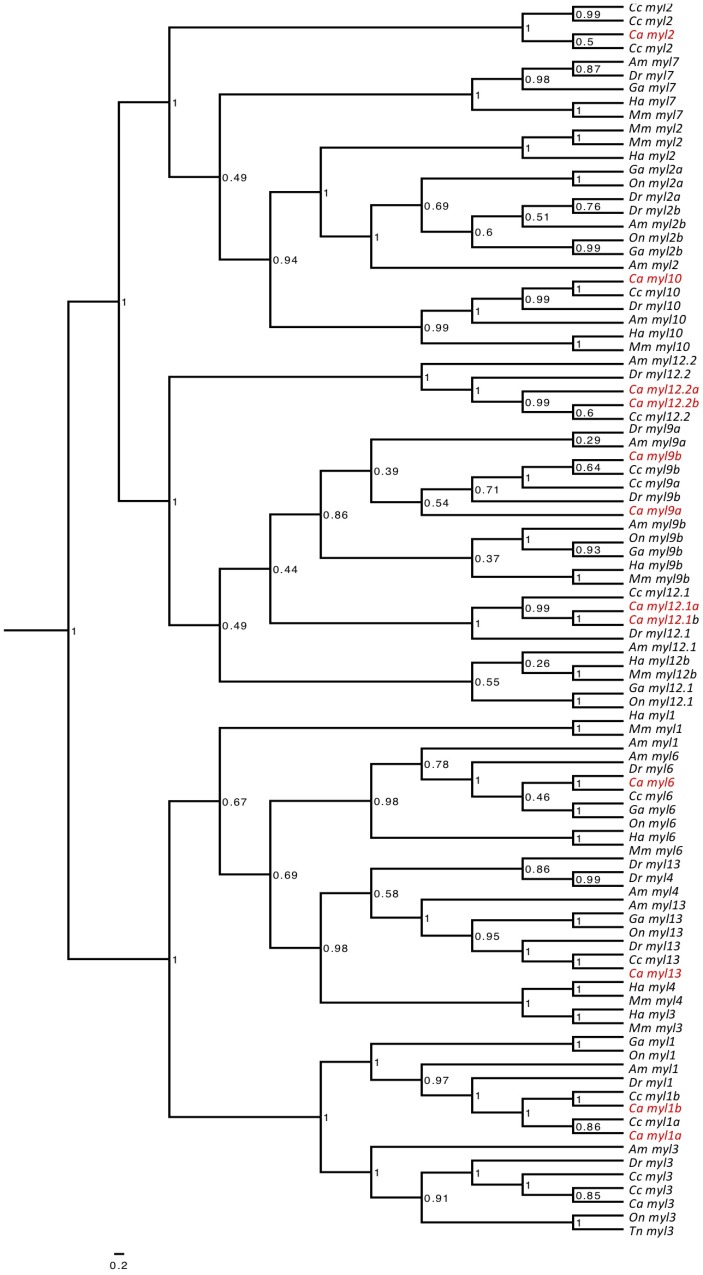
Phylogenetic tree constructed for myosin light chain genes. Bayesian phylogenetic relationships for the myosin light chain orthologs. Phylogenetic tree node numbers represent posterior values. To ease interpretation, goldfish orthologs (*Carassius auratus, Ca*) are highlighted in red. Myosin heavy chain orthologs for zebrafish (*Danio rerio, Dr*), stickleback (*Gasterosteus aculeatus, Ga*), common carp (*Cyprinus carpio, Cc*), green spotted pufferfish (*Tetraodon nigroviridis, Tn*), tilapia (*Oreochromis niloticus, On*), cavefish (*Astyanax mexicanus*, *Am*), medaka (*Oryzias latipes, Ol*) and human (*Homo sapiens, Ha*) were also included.

13 myosin light chain (*myl*) genes were identified: *myosin light chain 1a* (*myl1a*), *myosin light chain 1b* (*myl1b*), *myosin light chain 2* (*myl2*), *myosin light chain 9a* (*myl9a*), *myosin light chain 9b* (*myl9b*), *myosin light chain 3* (*myl3*), *myosin light chain 13* (*myl13*), *myosin light chain 6* (*myl6*), *myosin light chain 12.1a* (*myl12.1a*), *myosin light chain 12.1b* (*myl12.1b*), *myosin light chain 12.2a* (*myl12.2a*), *myosin light chain 12.2b* (*myl12.2b*), and *myosin light chain 10* (*myl10*) (Supplementary File S4). The abundantly expressed sequences *myl2*, *myl3*, *myl1a*, and *myl1b* clustered *in* monophyletic branches with their zebrafish orthologs following phylogenetic analysis (Figure 2).

### Myosin Heavy but Not Light Chain Transcripts Are Differentially Regulated With Temperature Acclimation

**FIGURE 3:**
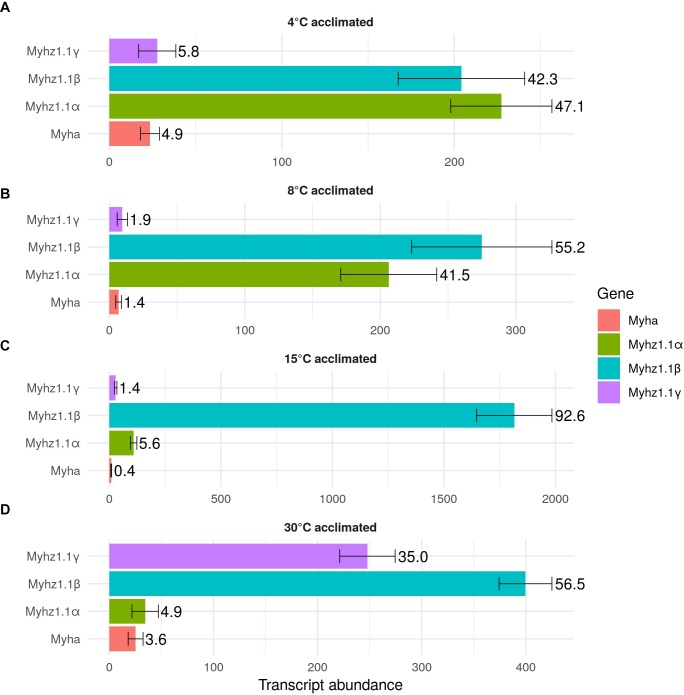
Transcript abundance of the main myosin heavy chain transcripts in goldfish fast skeletal muscle. Values represent Mean ± *SD* transcript copy number normalized to the reference gene. The transcripts of myosin heavy chain orthologs *myhz1.1α* (green), *myhz1.1β* (blue), *myhz1.1γ* (purple), and *myha* (red) in response to long-term acclimation to 4, 8, 15, and 30°C are illustrated. To ease interpretation, the percentage of each transcript is shown to the right of each bar. **(A)** 4°C acclimated, **(B)** 8°C acclimated, **(C)** 15°C acclimated and **(D)** 30°C acclimated.

**FIGURE 4:**
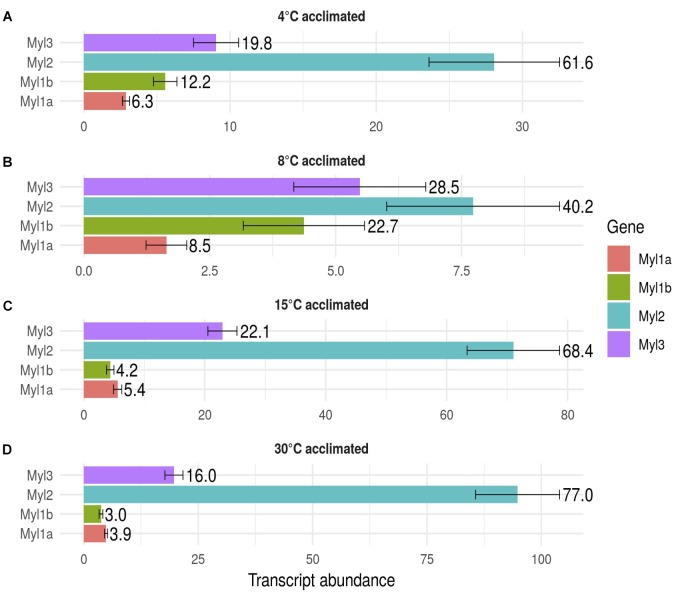
Transcript abundance of the main myosin light chain isoforms in goldfish fast skeletal muscle. Values represent Mean ±*SD* transcript copy number normalized to the reference gene. The transcripts of myosin light chain orthologs *myl1a* (red), *myl1b* (green), *myl2* (blue), and *myl3* (purple) in response to long-term acclimation to 4, 8, 15, and 30°C are illustrated. To ease interpretation, the percentage of each transcript is shown to the right of each bar. **(A)** 4°C acclimated, **(B)** 8°C acclimated, **(C)** 15°C acclimated and **(D)** 30°C acclimated.

Four *myh* (*myhz1.1α*, *myhz1.1β*, *myhz1.1γ*, and *myha*) and four *myl* (*myl2*, *myl3*, *myl1a*, and *myl1b*) constituted > 96% of the transcripts expressed for these proteins in fast myotomal muscle. Total transcript copy number for the four abundantly expressed *myh* transcripts showed a distinct optimum in fish acclimated to 15°C for 12 weeks and were 63% lower in the 30°C group and 75% lower at 4 and 8°C (*P* < 0.01) (Figure 3). *Myhz1.1α* and *myhz1.1β* comprised 90, 96, and 97% of *myh* transcripts at 4, 8, and 15°C, falling to 62% at an acclimation temperature of 30°C. The ratio of *myhz1.1α* and *myhz1.1β* transcripts was similar in the 4 and 8°C acclimation groups whereas *myhz1.1β* was 17 and 12 times more highly expressed in 15 and 30°C acclimation groups relative to *myhz1.1α* (*P* < 0.01) (Figures 3B,C). The *myhz1.1γ* transcript copy number was a minimum of nine-fold higher in the 30°C-group than at any other acclimation temperature (*P* < 0.01) (Figure 3D). The copy number for *myha* was much lower than the other more abundant *myh* transcripts and showed no consistent pattern with acclimation temperature (Figure 3). Each myosin heavy chain peptide is associated with two myosin light chain peptides, but this was not reflected in relative transcript abundances. For example, total transcript abundance for myosin light chains was lower at acclimation temperatures of 4 and 8°C than at 15 and 30°C (Figure 4). In contrast to myosin heavy chains the relative proportions of transcripts for the different orthologs of myosin light chains was largely independent of acclimation temperature (Figure 4).

## Discussion

The advent of genome sequencing and RNA-seq has illustrated the extraordinary diversity of myosin heavy and light chain genes that are expressed even in single muscle fiber types of vertebrates (Lynch et al., 2015). In the present study, 11 myosin heavy chains (*myh*) and 13 myosin light chain (*myl*) genes were identified in myotomal samples that only contained fast twitch muscle fibers. Just four transcripts (*myhz1.1α*, *myhz1.1β*, *myhz1.1γ*, and *myha*) comprised more than 96% of all the expressed myosin heavy chains. Myotubes are continuously produced in the fast myotomal muscle of teleost fish until 44% of the maximum adult body length (Weatherley et al., 1988). *In situ* hybridization studies in zebrafish have shown that myotubes/immature muscle fibers express five developmental-stage specific myosin heavy chain genes which become downregulated as fiber diameter and body size increase (Johnston et al., 2009). Since juvenile goldfish were used in our study some of the minor myosin heavy chain components e.g., *emb1_myh* probably corresponded to transcripts expressed in differentiating fast muscle myotubes. Other minor components were transcripts highly expressed in slow twitch or cardiac muscle (Supplementary File S4). For example, *smyh2* and *smyh3* are paralogs of human MYH7 which is highly expressed in slow-twitch muscle (Elworthy et al., 2008; Naganawa and Hirata, 2011) and *myha* is an trunk, tail, and cranial muscles (Nord et al., 2014). Although myofibers constitute the dominant component of fast muscle it also contains capillaries and motor neurones as well as other cell types such as macrophages, all of which express myosin heavy chain genes. For example, we detected *myh9* and *myh10*, non-muscle myosin heavy chains, which are thought to play a role in cytokinesis (Yang et al., 2012) and *myh11* a myosin heavy chain highly expressed in smooth muscle (Wallace et al., 2005).

The Cyprinidae family including goldfish, common carp, and the killifish *Fundulus heteroclitus* all show large increases in maximum swimming performance at low temperatures following a period of several weeks cold-acclimation at the expense of reduced performance at warm-acclimation temperatures (Fry and Hart, 1948; Johnson and Bennett, 1995). Seasonal plasticity of swimming performance in these species reflects a profound and complex remodeling of muscle phenotype at the level of proteins and organelles which serve to modify twitch contraction times, force production, shortening speed, and metabolic characteristics according to the prevailing environmental conditions (reviewed in Johnston and Temple, 2002). Differential expression of myosin heavy chains, but not myosin light chains, at the transcript and protein levels has been shown to be an important component of the mechanism underlying adjustments in myofibrillar ATPase activity and contractile properties in goldfish (Johnson and Bennett, 1995) and common carp (Imai et al., 1996). In *Fundulus heteroclitus* which is unable to modify myosin heavy chain composition with temperature acclimation much more modest adjustments in twitch contraction times and myofibrillar ATPase activity were observed (Johnson and Bennett, 1995). The short-horned sculpin (*Myoxocephalus scorpius*) of the family Cottidae showed a modest increase in the maximum shortening speed of fast muscle fibers at 15°C following acclimation from 5 to 15°C for 1–2 months, but unlike Cyprinidae, contractile performance (Ball and Johnston, 1996) and swimming behavior (Temple and Johnston, 1998) were unaffected by cold acclimation. Increased maximum muscle shortening speed of fast fibers with warm acclimation in short-horned sculpin was associated with altered expression of myosin light chains independently of myosin heavy chain composition (Ball and Johnston, 1996). These results indicate considerable species diversity in the response of the musculoskeletal system to temperature acclimation at the whole animal, tissue and molecular levels. However, a transition to a more aerobic phenotype is generally observed in all species following cold acclimation regardless of whether or not this is accompanied by altered locomotory performance (Johnston and Maitland, 1980; Egginton and Sidell, 1989; McClelland et al., 2006).

In our study on goldfish the total copy number of the most abundantly expressed myosin heavy chain transcripts showed a distinct optimum at 15°C with the relative proportions of *myhz1.1α*, *myhz1.1β, and myhz1.1γ* transcripts differing markedly in proportion above or below this temperature. Acclimation to temperatures at or above 15°C resulted in a marked increase in the relative expression of *myhz1.1β* and *myhz1.1γ* at the expense of *myhz1.1α.* The sequences of *myhz1.1α*, *myhz1.1β*, and *myhz1.1γ* in goldfish are orthologous to the 10, 15, and 30°C type myosin heavy chain genes described in the common carp according to the temperature at which they were expressed (Imai et al., 1996). Goldfish and common carp cluster on a common lineage of the Cyprinidae phylogenetic tree which split after the lineage leading to zebrafish (Huang et al., 2017). Our analysis shows that the three goldfish *myhz1.1* paralogs arose through the duplication of a single *myhz1.1* found in zebrafish which is a more basal cyprinid which has a more modest temperature range of 15 to 30°C and shows a shift to a more aerobic phenotype with cold acclimation, but no ability to modify maximum swimming speed (McClelland et al., 2006).

Local duplications seem to be common in cyprinids, for example zebrafish have three copies of the myhz1 (*myhz1.1*, *myhz1.2*, and *myhz1.3*), three copies of the slow myosin heavy chain (*smyh1*, *smyh2*, and *smyh3*) and several copies of cryptochrome genes all of them originated by local duplication (McGuigan et al., 2004; Liu et al., 2015). There is also evidence of a whole genome duplication event (WGD) event occurring on the cyprinids lineage 8 Million years ago (Li et al., 2015). Because we cannot distinguish between local duplication and whole genome duplication from the current analysis we named the duplicated genes as α, β, and γ following the annotation method suggested by Garcia de la serrana and Macqueen (2018). The medaka *Oryzias latipes*, can also modify myosin heavy chain expression following acclimation to either 10 or 30°C. In this species, luciferase reporter constructs involving deletions and site mutations of the 5′ flanking region of the gene established that a MEF2 transcription factor binding site was essential for expression in an acclimation-temperature dependent manner (Liang et al., 2008). Together these results indicate that a single duplication event followed by sub-functionalization may have been responsible for the extreme ability of these Cyprinidae species to modify maximum swimming with seasonal temperature acclimation.

## Author Contributions

DG and KW were responsible for animal maintenance, sampling, and data collection. DG was responsible for gene expression analysis. IJ and DG planned the experimental procedures and wrote the manuscript.

## Conflict of Interest Statement

The authors declare that the research was conducted in the absence of any commercial or financial relationships that could be construed as a potential conflict of interest.

## Footnotes

1 ensembl.org

2 ncbi.nlm.nih.gov

3 www.fishbrowser.org

## Supplementary Material

FILE S1 Length and weight of experimental animals.

FILE S2 Sequences used for phylogenetic reconstruction of myosin heavy chains.

FILE S3 Primers used for Sequencing and qPCR analysis.

FILE S4 Goldfish myosin heavy chains and myosin light chains sequences reported.
