# Supplementary material for: Duplication of a Single myhz1.1 Gene Facilitated the Ability of Goldfish (Carassius auratus) to Alter Fast Muscle Contractile Properties With Seasonal Temperature Change
Source: Front Physiol. 2018 Dec 4;9:1724. doi: 10.3389/fphys.2018.01724 (PMC6290348; doi:10.3389/fphys.2018.01724)
Supplement: FILE S1 — Length and weight of experimental animals. [file Table_1.DOCX]

**Supplementary File S1 Length and weight of experimental animals**

|  | 4^o^C | 8^o^C | 15^o^C | 30^o^C | p-val |
| --- | --- | --- | --- | --- | --- |
| Weight (g) | 16.56±0.28 | 17.01±0.78 | 17.44±0.78 | 19.61±0.91 | 0.58 |
| SL (cm) | 7.92±0.07 | 7.95±0.11 | 7.95±0.10 | 7.98±0.11 | 0.57 |
| FL (cm) | 9.12±0.09 | 9.08±0.12 | 9.20±0.16 | 9.34±0.13 | 0.71 |

SL: Standard length; FL: Fork length. Values indicate mean±SD (N=20).
