# Supplementary material for: Duplication of a Single myhz1.1 Gene Facilitated the Ability of Goldfish (Carassius auratus) to Alter Fast Muscle Contractile Properties With Seasonal Temperature Change
Source: Front Physiol. 2018 Dec 4;9:1724. doi: 10.3389/fphys.2018.01724 (PMC6290348; doi:10.3389/fphys.2018.01724)
Supplement: FILE S2 — Sequences used for phylogenetic reconstruction of myosin heavy chains. [file Table_2.DOCX]

Supplementary File S2

SEQUENCES USED FOR PHYLOGENETIC RECONSTRUCTION OF MYOSIN HEAVY CHAINS

>ENSDARG00000099959_danio_smyhc1

>ENSDARG00000103969_smyhc2_danio

>ENSONIG00000008086_oreochormis_smyhc1

>ENSGACG00000002145_gasterosteus?smyhc1

>ENSTNIG00000003838_tetraodon_smyhc1

>ENSAMXG00000012723_smhyc1_astyanax

>ENSG00000092054_homo_MYH7

>ENSDARG00000090637_danio_myhc6

>ENSGACG00000013782_gasterosteus_myhc6

>ENSONIG00000021261_oreochromis_myhc6

>ENSDARG00000012944_danio_myhz2

>ENSDARG00000035438_danio_mhyc4

>ENSDARG00000095930_danio_myhca

>ENSDARG00000067995_myhz1.2

>ENSDARG00000067997_danio_myhz1.3

>ENSGACG00000002933_gasterosteus_myha

>ENSGACG00000003003_gasteorsteus_myhca_2_2

>ENSONIG00000011145_oreochromis_myhz2

>ENSTNIG00000006766_tetraodon_myhz2

->ENSORLG00000013060_medaka_myhz2

>D89992.1| Cyprinus carpio

>D89990.1| Cyprinus carpio

>D89991.1| Cyprinus carpio

>ENSDARG00000001993_danio_myhb

>ENSGACG00000010000_gasterosteus_myhc2.1

>ENSGACG00000010018_gasterosteus_myh2.2

>ENSTNIG00000007015_tetraodon_myhc2

>ENSONIG00000001739_oreochromis_myhc2

>ENSGACG00000001528_gasterosteus_myhb_2_2

>ENSTNIG00000014702_tetraodon_myhcb_1_2

>ENSTNIG00000014704_tetraodon_myhcb_2_2

>ENSG00000109061_homo_MYH1

>ENSG00000264424_homo_MYH4

>ENSG00000125414_HOMO_MYH2

>ENSG00000133020_myh8_Homo

>ENSDARG00000001993_danio_myhb

>ENSGACG00000001522_gasterosteus_myhb_1_2

>ENSORLG00000013060_medaka_myhz2

>ENSDARG00000102414_myhz1.1_danio

SEQUENCES USED FOR PHYLOGENETIC RECONSTRUCTION OF MYOSIN HEAVY CHAINS

>ENSDARG00000104722_danio_mlc2a

>ENSDARG00000053424_danio_mlc2b

>ENSAMXG00000006106_astyanax_mlc2b

>ENSMUSG00000013936_mus_mlc2

>ENSGACG00000008592_gasterosteus_mmlc2b_1_2

>ENSGACG00000013491_gasterosteus_mlc2b_2_2

>ENSONIG00000015965_oreochormis_mlc2b

>ENSAMXG00000006952_astyanax_mlc2

>ENSONIG00000019442_oreochromis_mlc2

>ENSDARG00000062592_danio_mlc10

>ENSAMXG00000008806_astyanax_mlc10

>ENSDARG00000019096_danio_mlc7

>ENSAMXG00000015566_astyanax_mlc7

>ENSGACG00000004447_gasterosteus_mlc7

>ENSDARG00000038123_danio_mlc9a

>ENSAMXG00000001054_astyanax_mlc9a

>ENSGACG00000008160_gasterosteus_mlc9b

>ENSONIG00000019403_oreochromis_mlc9b

>ENSDARG00000008030_astyanax_mlc9b

>ENSAMXG00000015977_astyanax_mlc9b

>ENSDARG00000099766_danio_mlc12.1

>ENSAMXG00000011297_astyanax_mlc12.1

>ENSGACG00000004252_gasterosteus_mlc12.1

>ENSONIG00000006115_oreochormis_mlc12.1

>ENSDARG00000025326_danio_mlc12.2

>ENSAMXG00000012366_astyanax_mlc12.2

>ENSDARG00000014196_danio_myl1

>ENSAMXG00000006751_astyanax_myl1

>ENSGACG00000006539_gasterosteus_myl1

>ENSONIG00000004223_oreochormis_myl1

>ENSDARG00000017441_danio_mlc3

>ENSAMXG00000009232_astyanax_mlc3

>ENSONIG00000013405_oreochromis_mlc3

>ENSTNIG00000008325_tetraodon_mlc3

>ENSDARG00000011519_danio_myl4

>ENSAMXG00000019470_astyanax_myl4

>ENSDARG00000099712_danio_myl3

>ENSDARG00000042245_danio_myl13

>ENSAMXG00000010314_astyanax_myl13

>ENSGACG00000017400_gasterosteus_myl13

>ENSONIG00000009463_orechromis_myl13

>ENSDARG00000008494_danio_myl6

>ENSAMXG00000001694_astyanax_myl6

>ENSGACG00000000696_gasterosteus_myl6

>ENSONIG00000019103_oreochromis_myl16

>ENSG00000168530_homo_myl1

>ENSMUSG00000061816_mus_myl1

>ENSG0000011124_homo_myl2

>ENSMUSG00000013936_mus_myl2

>ENSG00000092841_homo_myl6

>ENSMUSG0000009084_mus_myl6

>ENSG00000198336_homo_myl4

>ENSMUSG00000061086_mus_myl4

>ENSG00000106631_homo_myl7

>ENSMUSG00000020469_mus_myl7

>ENSG00000106436_homo_myl10

>ENSMUSG00000005474_mus_myl10

>ENSG00000101335_homo_myl9

>ENSMUSG00000067818_mus_myl9

>ENSG00000118680_homo_myl12B

>ENSMUSG00000034868_mus_myl12B

>ENSG00000160808_homo_myl3

>ENSMUSG00000059741_mus_myl3
