# Supplementary material for: Duplication of a Single myhz1.1 Gene Facilitated the Ability of Goldfish (Carassius auratus) to Alter Fast Muscle Contractile Properties With Seasonal Temperature Change
Source: Front Physiol. 2018 Dec 4;9:1724. doi: 10.3389/fphys.2018.01724 (PMC6290348; doi:10.3389/fphys.2018.01724)
Supplement: FILE S3 — Primers used for Sequencing and qPCR analysis. [file Table_3.DOCX]

Supplementary File S3

| Protocol | Gene name | Forward | Reverse |  |  |
| --- | --- | --- | --- | --- | --- |
| qPCR | Myhz1.1b | GAGTCCAGGGTGCATGAGTT | GATCCTGCAGTCGAGTCACA |  |  |
|  | Myhz1.1c | ACAAGGACCCACTGAACGAC | GGAAGGAACCACCCTTCTTC |  |  |
|  | Myhz1.1a | ATGACCTGGAGCTCACCTTG | AGTCTGCTGGTGTGCCTCTT |  |  |
|  | Myhb | GGCAAACTGATGACCAACCT | GCAAATTCTGATTCCCTCCA |  |  |
|  | Myl2 | ATGGTGAGGAAAACGGTGAA | CATGTTTGAGCAGAGCCAGA |  |  |
|  | Myl3 | GCTGTTGTAACCCTGGATGG | CGCCCACCTTTTTGAAGTTA |  |  |
|  | Myl1a | CCTTGTTTGGGCTGTTGACC | CAACCCTACCGCCGATGACATGG |  |  |
|  | Myl1b | AAGGAGGGTTTTCAGCCAGT | GGACAAGGCTCTCCGTAGC |  |  |
|  | RPL27 | GTCTCCGCCAGATCTTCAAC | GGCAGTTGTCTGTCAGTGGA |  |  |
|  |  |  |  |  |  |
| Sequencing | Smyhc2 | AACTGAGGCTCCTCCTCACA | ATCCTTCTTGGTTGGTGCTG |  |  |
|  | Smyhc3 | AGCAGACCATCAAGGACCTG | CTCTGTTCCAGCTCCACCTC |  |  |
|  | Myhz1.1a | GGCTTCACTGCTGATGAGAAAATC | CCGACCTTCACTCTGGGGTA |  |  |
|  | Myhz1.1b | GGATGAGGCTGAGAGTCTGG | CCGAGCCACATTCTTCTTGT |  |  |
|  | Myhz1.1c | GCAACTGTTGGTGCAATGTC | GATGCCAGTTTTCCAGTGGT |  |  |
|  | Myhcb | GGCAAACTGATGACCAACCT | GCAAATTCTGATTCCCTCCA |  |  |
|  | Myhca | AAGCCAGGCTGAACTAGAAGG | CCAAGCTGCTCAGAGAGGTC |  |  |
|  | Emb1_myh | GGTCTGCTGATGTGCCTCTT | TGATGACCTGGAGCTGACCT |  |  |
|  | Myh9b | CCAGAGCAGCTCGGTCCT | GTCTGGGCCTTCTGCACATA |  |  |
|  | Myh10 |  |  |  |  |
|  | Myh11 | AGCTCCAGGCTCAAATGAAA | CCTCACTCTCCTCCAACTGC |  |  |
|  | Myl2 | ATGGTGAGGAAAACGGTGAA | CATGTTTGAGCAGAGCCAGA |  |  |
|  | Myl12.1 | TCGTCGACCTCTTCGTCTG | GTTCGGAGAGAAGCTGAACG |  |  |
|  | Myl12.2 | CAGTAGTGCTGGGCCTTGTC |  |  |  |
|  | Myl3 | GCTGTTGTAACCCTGGATGG | CGCCCACCTTTTTGAAGTTA |  |  |
|  | Myl1a | CCTTGTTTGGGCTGTTGACT | CCATATCATCAGCTGTAGGGTTG |  |  |
|  | Myl1b | AAGGAGGGTTTTCAGCCAGT | AAGGAGGGTTTTCAGCCAGT |  |  |
|  | Myl13 | TTGGAACGCATCCTTGAAAT | CCCCCAAGAAGAAGGAAGAG |  |  |
|  | Myl9a | AACCCTGCGGTCAGAATGT | CAGACGTTCTCCAAACATGG |  |  |
|  | Myl9b | CCTGAAACTCCGTCATCATGT | GCTGACGCTCTCTGATCATCT |  |  |
|  | Myl6 | ACACACACCCGGAAAACAAG | TGTACTTTATTGGACAATGAGGATATT |  |  |
|  | Myl10 | TTCATCAAGCTCATCGTTGC | TGCCAGCTCCAATGTGTTTA |  |  |
