# Supplementary material for: Duplication of a Single myhz1.1 Gene Facilitated the Ability of Goldfish (Carassius auratus) to Alter Fast Muscle Contractile Properties With Seasonal Temperature Change
Source: Front Physiol. 2018 Dec 4;9:1724. doi: 10.3389/fphys.2018.01724 (PMC6290348; doi:10.3389/fphys.2018.01724)
Supplement: FILE S4 — Goldfish myosin heavy chains and myosin light chains sequences reported. [file Table_4.DOCX]

Supplementary File S4

>slow_myosin_heavy_chain_2_(smyhc2)

CAAGCTCACATCATTGTACCACCTAAAGGACTTGAACTTTTATTGGATTTTATCTGTCTGACTGAAAGATGGGGGACGCCCTGATGGCAGAGTTCGGACCTGCTGCTTCCTTTCTGCGTAAGTCAGACAAGGAGCGCCTGGAGGCCCAGACTCGCATCTTCGACATGAAGAAGGAATGCTTTGTGCCTGATGCTGAGGTTGAGTACGTCAAAGCCTCTATCACCAGTAGAGACGGTGACAAAGTCACTGTTAACACTGAATTTGGAAAGACAGTAACTGTGAAGGACATTGATGTTCATCCACAAAACCCGCCAAAGTTTGATAAAATTGAGGACATGGCGATGTTCACCTTCCTGCATGAGCCCGCTGTGCTGTTTAACCTCAAAGAGCGTTACGCAGCTTGGATGATCTACACCTACTCTGGGCTCTTCTGTGTCACTGTCAACCCCTACAAGGGGCTACCAGTGTACAACCAGGAAGTGGTCACTGCCTACAGAGGAAAGAAACGAACTGAGGCTCCTCCTCACATCTTCTCCATCTCTGACAACGCCTACCAATACATGCTGTCAGACAGAGAAAACCAGTCTGTCCTGATCACTGGAGAATCCGGTGCTGGAAAGACTGTGAACACTAAAAGAGTCATCCAGTACTTTGCCAGCATTGCTGCAGCACCAACCAAGAAGGATGCATCAATGGAGAAAAAGGGAACTCTGGAGGATCAAATCATCCAGTGTAATCCTGCTCTGGAGGCTTTTGGTAATGCCAAGACCATTAGAAATGACAACTCTTCCAGATTCGGTAAATTTATCCGTATCCATTTTGGAATCAGTGGAAAGTTAGCTTCTGCTGACATTGAGACTTATCTGCTGGAGAAGTCTCGCGTCACTTTTCAGCTCAAGGCTGAGAGAGACTACCACATCTTCTACCAGATCCTCTCCCAGAGAAAACCAGAACTGCTAGAGATGCTGCTCATCACCAACAACCCCTATGACTACTCCTTCATCTCCCAAGGAGAGACACAAGTGCAATCTATTGATGACCGTGACGAGCTGATGGCCACTGATGAAGCATTTGATGTGCTGGGCTTCACTACAGATGAAAAGGCAGGCATCTACAAGCTGACTGGTGCTGTCATGCACTACGGCAACATGAGGTTCAAGCAGAAGCAGCGAGAGGAACAGGCAGAGGCTGATGGGACTGAGGATGCTGACAAAGTCGCATATCTGATGGGTCTGAACTCTGCTGATCTCATCAAGGGTTTGTGCCACCCGAGAGTCAAAGTAGGAAATGAGTGGGTCACCAAGGGACAGAGTGTCCAACAGGTGTACTACTCTATTGGTGCTCTGGCAAAGTCAGTGTACGAGAAGATGTTCTTGTGGATGGTTGTGAGAATCAACCAATCCCTGGACACCAAACAGCCTCGCCAGTACTTCATTGGTGTGCTGGACATTGCTGGATTTGAGATCTTTGATTACAACACGTTTGAGCAGCTGTGCATCAACTTCACTAATGAGAAGTTGCAGCAGTTCTTCAACCATCACATGTTTGTTCTGGAGCAAGAGGAATACAAGAAGGAGGGTATTGAATGGGTGTTCATTGACTTTGGCATGGACTTAGCGGCTTGTATTGAGTTAATTGAGAAGCCCATGGGTATCATGTCCATCCTTGAAGAGGAGTGCATGTTCCCAAAAGCCAGTGATGCAACATTCAAATCTAAGCTTTATGACAACCACTTGGGCAAAAGTCCTAACTTCCAGAAGCCCAGGATTGTCAAGGGTAAACCAGAGGCGCATTTCTCCCTGGTTCACTACGCTGGCATTGTTGACTACAACATCTCAAACTGGCTGGTGAAGAACAAGGATCCTCTCAATGAGACGGTTGTAGGGTTGTTCCAGAAGTCCACCATGAAACTGCTTTCTGTCCTATTTGCTAATTATGCTGCTAGTGATGCAGATTCTGGAGCTAAGAGTGCCAAAGGAGGTACCAAAAAGAAGGGTTCTTCATTCCAAACAGTGTCAGCCCTCCATAGGGAGAACCTGCACAAGCTCATGACCAACTTGAAGTCAACTCACCCTCATTTTGTGCGTTGCCTGATTCCCAATGAGACTAAGACTCCTGGGGCGATGGAGAATCCTCTGGTCATGCACCAGCTGCGCTGTAACGGTGTGCTGGAGGGCATCAGAATCTGCACAAAGGGCTTCCCCAACAGGATCCTGTATGGAGATTTCAAACAACGGTACCGGATCCTAAATCCAGCGGCTATCCCTGAGGGACAGTTCATTGATAATAAGAAGGGTGCAGAAAAGCTTCTGGGTTCTCTTGACATTGATCACAGCCAGTACAAGTTAGGACACACTAAGGTGTTCTTCAAGGCTGGTCTTCTGGGAACTCTTGAAGAGATGAGAGACGACCGTCTTGCACTTATTATTACTGGAATTCAGTCCAGATCTCGTGGTTTTCTATCAAGAATGGAATTCCAGAAAATTGTGGAGAGAAGAGATGCCTTGCTTGTGATTCAGTGGAACATCCGTGCCTTCATGGGGGTCAAGAATTGGCCTTGGATGAAGCTCTACTTCAAGATCAAGCCTCTGCTGAAATCTGCTGAGACTGAGAAAGAGATGGCCAACATGAAGGAAGAATTCCTGAAGTTGAAGGAGGTTTACGCCAAAACTGAAGCTCGCAGAAAGGAGCTTGAGGAAAAGATGGTTTCCATTCTCCAAGAGAAGAATGACCTTCAGCTTGCAATGCAGTCTGAGCAAGACAATCTTGCAGATGCTGAGGAGAGATGTGAGGGTCTGATCAAGAGCAAGATTCAGTTTGAAGGTAAAGTCAAAGAAATGACTGAGAGACTGGAGGATGAAGAGGAAATGAATGCTGAGCTGACTGCCAAGAAACGAAAGCTGGAGGATGAATGTTCTGAGCTCAAGAAGGACATTGATGATCTTGAGCTCACTCTGGCCAAAGTGGAAAAAGAGAAACATGCCACTGAGAACAAGGTTAAAAACCTGACAGAAGAGATGGCAGCATTGGATGAGATCATTGCTAAGCTGACCAAAGAGAAGAAAGCTCTGCAGGAGGCCCATCAGCAAACACTGGATGACCTCCAGAGTGAGGAAGACAAAGTCAACACACTGACCAAAGCCAAAGCCAAGCTGGAGCAACAAGTGGATGATCTTGAAGGTTCTCTGGAACAAGAAAAGAAAATTCGCATGGATCTTGAGAGAGTTAAAAGAAAGCTTGAGGGTGACTTGAAGTTGACTCAAGAAAATTTGATGGATTTAGAAAATGATAAGCAGCAGATGGATGAGCGGATAAAGAAAAAAGATTTTGAGATCAGCCAGCTCAACAGCAAGATTGAAGATTGTCAGGTTATAGAATCCCAACTCCAGAAGAAACTGAAGGAGCTGCAGGCCCGAATTGAAGAGCTGGAGGAAGAGCTGGAGGCTGAGAGAGCCGCTCGTGCCAAAGTTGAGAAACAGAGAGCTGATCTGTCCAGAGAACTGGAGGAGATCAGTGAGAGGCTGGAGGAGGCTGGTGGAGCCACTGCTGCCCAGGTTGAAATGAACAAGAAACGTGAAGCCGAGTTACAGAAGCTGCGTAGAGACCTTGAAGAGGCCACTCTGCAACACGAGGCTACCGCTGCTACACTGAGGAAGAAACATTCTGACAGTGTGGCCGATCTGGGAGAACAGATTGACAACCTTCAGAGAGTGAAGCAGAAGCTGGAGAAAGAGAAGAGTGAACTCAGACTGGAACTGGATGATGTGGTCTCCAACATGGATCAGCTTGTAAAGGCCAAGGCAAATCTGGAGAAATTGTGCAGAACTCTGGAAGACCAGATGAGTGAGTACAGAACCAAGAGTGAGGAAGGCCAGCGCACAATCAATGACTTCACCATGCAAAAAGCCAAGCTGCAAACTGAGAATGGTGAACTGTCCAGACAGCTAGAGGAGAAAGACTCCCTGGTGTCTCAGTTGACCAGAGGCAAGCAGTCCTACACTCAGCAGGCTGAAGACCTCAAGAGACAGCTAGAGGAAGAGATCAAGGCTAAGAATGCCCTGGCCCATGCAGTTCAATCTGCTCGTCATGATTCTGATCTGCTGAGGGAACAGTACGAGGAGGAGCAGGAAGCCAAAGCTGAGCTGCAGCGGAGTCTGTCCAAGGCAAACTCTGAGGTGGCTCAGTGGAGAACTAAGTATGAAACTGATGCCATCCAGAGGACGGAAGAGCTGGAGGAAGCCAAGAAAAAACTGGCTCAGCGTCTGCAAGATGCAGAAGAAGCTGTGGAAGCAGTCAATGCTAAATGCTCCTCTCTGGAGAAGACCAAGCACAGGCTGCAGAATGAGATTGAAGATCTCATGGTGGATGTAGAGAGATCCAATGCTGCTGCCGCTGCTCTGGACAAGAAGCAAAGAAACTTTGACAAGGTTCTGGCTGAGTGGAAGCAAAAGTATGAGGAGTCCCAATCTGAATTGGAAAGTGCTCAAAAAGAAGCTAGATCTCTGAGCACTGAACTCTTTAAATTGAAAAACGCATATGAAGAGTCACTGGACCACCTGGAGACCATGAAGAGGGAGAACAAGAATCTCCAAGAGGAAATCTCTGACCTCACTGAGCAACTTGGTGAGAGTGGGAAGAATATTCATGAGCTGGAGAAACTTAGGAAACAACTGGAGCAGGAGAAACAAGAGATACAATCTGCTTTGGAGGAGGCTGAGGGTTCCCTTGAACATGAGGAAAGCAAGATCCTTAGAGCTCAGCTGGAATTCAATCAGATCAAAGCTGATATTGAACGTAAACTGGCTGAGAAAGATGAAGAGATGGAGCAGTCCAAGAGGAATCAGCAGAGAATGATTGATACCCTTCAGACCTCACTGGAATCAGAGACTCGCAGCAGGAATGAAGCTCTCAGACTGAAGAAGAAGATGGAGGGAGACCTCAATGAGATGGAGATTCAGCTCAGCCAGGCTAACAGACTGGCATCAGAATCTCAGAAACAACTCAAGGGACTTCATGGACATCTTAAAGACTCCCAACTGCAGCTGGATGATGCTCTGCGCAGTAATGATGATTTCAAAGAGAACATCGCAATTGTGGAGAGACGTAACAATCTGCTGCAGGCTGAACTGGATGAGCTGAGATCAATGGTGGAACAGACTGAGAGAGGAAGGAAACTGGCTGAGCAGGAACTGCTGGACATCAGTGAGAGAGTCCAGCTCCTGCATTCTCAGAATACCAGTCTGCTGAATCAGAAGAAGAAGCTGGAGGGAGATAATTCTCAGCTTCAGACTGAGGTGGAGGAAGCAGTGCAGGAGTGCAGGAACGCTGAGGAAAAAGCCAAGAAGGCCATCACTGATGCTGCCATGATGGCTGAGGAGCTGAAGAAGGAGCAGGACACCAGTGCTCACCTGGAGAGGATGAAGAAGAACATGGAGGTGACTGTCAAAGACCTACAGCACCGTCTGGATGAAGCTGAGCAGATCGCAATGAAGGGAGGCAAGAAGCAGGTCCAGAAATTGGAGGCCAGGGTGAGAGAGCTGGAAAGTGAGGTGGAGATAGAACAGAGAAAGGCCAGTGACTCTGTCAAGGGGATTCGTAAATATGAAAGACGCATCAAAGAACTCACCTACCAGACTGAGGAGGACCGTAAGAATCTGGCTCGCCTGCAGGACTTGGTGGACAAACTCCAGCTGAAGGTCAAGTCCTACAAGAGAGTTGCTGAAGAGGCTGAAGAACAAGCAAATTCTAGCCTGGGCAAGTTCCGTAAGATCCAGCATGAGCTGGATGAAGCAGAGGAGAGGGCTGATATTGCTGAATCTCAGGTCAACAAGCTGAGAGTCAAGAGTCGTGATACAGGATCCAAGAAGGGGGCTGATGAGGAGTGA

>slow_myosin_heavy_chain_3_(smyhc3)

CCTCAGGAAGTCGGACATAGAGCGTCTGGAGGCCCAAACTCGCCCCTTTGACATGAAGAAAGCTTGTTTCGTTCCTGATGCTGAAGAGGAGTATGTGAAAGCAACTATTATTAGTCGAGATGGTGATAAAGTCACCTGTGAGAATTCAAAGGGAACGACTTTAACTGTGAAAGAAGTGGATGTTCATCCTCAGAACCCGCCAAAGTTTGATAAAATTGAGGACATGGCGATGTTCACCTTCCTGCATGAGCCCGCTGTGCTGTTTAACCTCAAAGAGCGTTACGCAGCCTGGATGATCTACACCTACTCAGGGCTCTTCTGTGTCACTGTCAACCCCTACAAGTGGCTCCCGGTGTACAACCAGGAAGTGGTCGTTGCCTACAGAGGAAAGAAGAGGAGTGAAGCTCCTCCTCACATCTTCTCCATCTCTGACAACGCCTACCAATACATGCTGTCAGACAGAGAAAACCAGTCCATTCTTATCACTGGAGAATCTGGTGCTGGAAAGACTGTGAACACCAAGAGAGTCATTCAGTACTTTGCCAGCATTGCTGCTAGTCCTTCAAAGAAGGAAACCACTGAAAAGAAGGGAACTCTGGAGGATCAAATCATCCAGTGTAATCCTGCTCTCGAGGCCTTTGGTAATGCCAAGACCATCAGAAATGACAACTCATCTAGATTTGGCAAGTTTATCCGGATCCACTTTGCTGCCAGTGGCAAACTAGCATCTGCTGATATTGAGACTTATCTTCTGGAGAAGTCTCGTGTGACTTTCCAGCTCAAGGCTGAGAGAGACTACCACATCTTCTACCAGATCCTGTCTCAGAAGAAACCAGAACTATTAGAGATGCTGCTGATCACAGCAAACCCTTATGATTATGCTTTCATCTCTCAAGGAGAGACACAAGTGGCCTCTATTAATGATGCTGATGAGCTGATGGCAACAGATGAAGCGTTTGATGTGTTGGGCTTCACCCAAGAGGAGAAGAACAGCATCTACAAGCTGACTGGTGCTGTCATGCACTACGGAAACATGAGGTTCAAGCAGAAGCAGCGAGAGGAACAGGCAGAGGCTGATGGGACTGAGGATGCTGATAAGTCAGCTTATCTGATGGGCCTGAACTCTGCTGATCTCATCAAGGCTCTGTGCCACCCGAGAGTCAAAGTAGGGAATGAGTGGGTCACCAAGGGACAGAATGTCCAGCAGGTGTCCTATGCCATTGGTGCTCTGTCAAAATCAGTGTACGAGAAGATGTTCCTCTGGATGGTTGTGAGAATCAACCAATCCCTGGACACCAAACAGCCTCGCCAGTACTTCATTGGTGTGTTGGACATTGCTGGATTTGAGATCTTTGATTTCAACACCTTTGAGCAGCTGTGCATCAACTTCACAAATGAGAAGTTGCAGCAGTTCTTCAACCATCACATGTTTGTTCTGGAGCAAGAGGAATACAAGAAGGAGGGTATTGAATGGGTGTTCATTGACTTTGGCATGGACTTGCAGGCTTGTATTGATCTTATTGAAAAGCCCATGGGTATCATGTCCATCCTTGAAGAGGAGTGCATGTTCCCCAAAGCCAGTGATGCAACATTCAAAGCTAAGCTTTATGACAATCACTTGGGCAAAAGTGCTAACTTCCAGAAGCCCAGGATTGTCAAGGGTAAACCAGAGGCACATTTCTCCCTGGTTCACTACGCTGGCACTGTTGACTACAACATCAATAACTGGCTGGTGAAGAACAAGGATCCTCTAAATGAGACGGTTGTTGGCCTGTATCAGAAATCCACAATGAAACTCCTTTCTATCCTATTTGCTAATTATGCTAGTGCTGACTCAGCCGCAGAGGGAGGTGGTGGCAAAGCAAAGGAGAAGAAGAAAAAGGGTTCTTCTTTCCAGACAGTGTCTGCTCTTCACAGGGAGAACCTGAACAAGTTGATGACCAACTTGAGGTCAACTCACCCTCACTTTGTGCGCTGCATCATCCCCAATGAGACTAAGACTCCTGGGGCGATGGAGAATCCTCTGGTCATGCACCAGCTGCGCTGTAACGGTGTGCTGGAGGGCATCAGAATCTGCAGAAAGGGCTTCCCCAACAGGATCCTGTATGGAGATTTCAAGCAGAGATATCGTATACTGAACCCTGCTGCCATCCCAGAGGGTCAATTCATTGACAGCAGAAAAGGAGCCGAGAAGTTATTGGGCTCTTTGGATATTGACCACAACCAGTACAAGTTTGGACATACTAAGGTGTTCTTCAAGGCTGGTCTTCTAGGTACTCTTGAGGAGATGCGAGATGACCGTCTTGCTCTCATCATCACAAATATTCAAGCTAGAGCTCGTGGTCTTCTCTCAAGAGTTGAGTTCCAGAAGATTGTTGAACGCAGAGATGCCTTGCTTGTGATCCAGTGGAATATCCGTGCCTTCATGGGTGTCAAGAATTGGCCCTGGATGAAGCTCTACTTCAAGATCAAGCCTCTTCTGAGATCTGCTGAAGCAGAGAAAGAAATGGCCAACATGAAGGAAGAATTCCTGAAGTTGAAGGAGGCTTACGCCAAATCTGAAGCCCGCAGAAAGGAGCTTGAAGAAAAGATGGTTTCTCTTCTCCAAGAGAAGAATGACTTGCAACTTCAAGTCCAAACTGAGCAAGATAATCTTTGCGATGCTGAGGAGAGATGTGAGGGTCTGATCAAGAACAAGATCCAGCTTGAGGCCAAAGCCAAAGAGCTGACTGAGAGACTGGAGGATGAGGAAGAAATGAATGCTGAGCTGACTGCCAAGAAACGAAAGTTGGAGGATGAATGTTCTGAGCTCAAGAAGGACATTGATGACCTTGAGCTCACTCTGGCCAAAGTGGAGAAAGAGAAACATGCCACTGAGAACAAGGTTAAAAACCTGACAGAAGAGATGGCAGCTTTGGATGAGATCATTGCTAAGCTGACCAAAGAGAAGAAAGCTCTGCAGGAGGCCCATCAGCAAACACTGGATGACCTCCAGAGTGAGGAAGACAAAGTCAATACACTCACCAAAGCCAAAGCCAAGCTGGAGCAACAAGTGGATGATCTTGAGGGTTCCCTGGAACAGGAAAAGAAGCTTCGTATGGACCTGGAGAGGGCAAAGAGGAAGCTCGAGGGAGACTTAAAGTTGACCCAAGAGAGCGTCATGGATCTGGAAAATGACAAACAGCAACTGGAAGAGAGGATTAAAAAGAAAGACTTTGAGATCAGCCAGCTCAACAGCAAGATTGAAGATGAGCAGGTTATGGCAGCCCAACTCCAGAAGAAACTGAAGGAGCTGCAGGCTCGGATTGAAGAGCTGGAGGAAGAGCTGGAGGCTGAGAGAGCCGCTCGTGCCAAAATTGAGAAACAGAGAGCTGATCTTTCCAGAGAACTGGAGGAGATCAGTGAGAGGTTGGAGGAGGCTGGTGGAGCCACTGCTGCCCAGATTGAGATGAACAAGAAACGTGAAGCCGAGTTACAGAAGCTGCGCAGAGACCTCGAAGAGGCCACTCTGCAACACGAGGCTACCGCTGCTACACTGAGGAAGAAACATTCTGACAGTGTGGCCGATCTGGGAGAACAGATTGACAACCTTCAGAGAGTGAAGCAGAAGCTGGAGAAAGAGAAGAGTGAACTCAGACTGGAACTGGATGATGTGGTCTCCAACATGGAGCAGCTTGCCAAGGCCAAGGCAAACTTGGAGAAAATGTGCAGGACCTTCGAGGACCAGATGTCAGAATATAGAACAAAATACGAAGAAGGACAACGTAGCATCAATGACTTTACCATGCAAAAAGCTAAATTGCAAACCGAAAATGGGGAGCTTTCTAGACAGCTGGAAGAGAAGGATTCCTTGGTCTCTCAGCTAACAAGAGGCAAGCAGTCCTACACTCAGCAGATTGAGGACCTCAAACGACAACTAGAGGAGGAAATTAAGGCTAAGAATGCCCTGGCCCCATGCAGTTCAATCTGCTCGTCATGATTCTGATCTGCTGAGGGAACAGTACGAGGAGGAGCAGGAAGCAAAAGCTGAGCTGCAGCGTAGTATGTCCAAAGCAAACTCTGAGGTGGCTCAGTGGAGAACTAAGTATGAAACTGATGCCATCCAGAGGACTGAGGAGCTGGAGGATGCTAAGAAGAAACTGGCTCAGCGTCTGCAAGATGCAGAAGAAGCTGTGGAAGCAGTCAATGCTAAATGCTCCTCTCTGGAGAAGACCAAGCACAGGCTGCAGAATGAGATTGAAGATCTCATGGTGGATGTGGAGAGATCCAATGCTGCTGCCGCTGCTCTGGACAAGAAGCAAAGAAACTTTGACAAGGTCCTGGCTGAATGGAAGCAGAAATATGAGGAGTCTCAGAGTGAGCTGGAAAGCTCCCAGAAGGAGGCCAGATCTCTGAGCACTGAACTCTTCAAACTGAAGAACTCCTACGAGGAGTCGCTGGATCATCTGGAGACCATGAAGAGAGAAAACAAGAACCTCCAAGAGGAGATCGCTGATCTCACTGAACAAATTGGTGAGAGTGGAAAGAACATTCATGAACTAGAGAAAGTTCGCAAACAACTGGAGCAGGAGAAAGCTGAAATTCAAGCAGCTTTGGAGGAGGCTGAAGGCTCACTTGAGCATGAAGAAGGCAAGATACTTAGAGCTCAACTGGAGTTCAATCAAGTAAAGGCTGATATTGAACGTAAGCTGGCTGAGAAAGATGAAGAGATGGAGCAGTCCAAGAGGAACCAGCAGAGAATGGTTGATACTCTGCAGAGCTCACTGGAATCAGAGACTCGCAGCAGGAATGAAGCTCTCAGATTGAAGAAGAAGATGGAGGGAGACCTCAATGAGATGGAGATTCAGCTCAGCCAGGCTAACAGACAGGCATCAGAAGCCCAGAAGCAACTCAAGGGTCTTCATGGACATCTTAAAGATTCCCAACTGCAACTGGATGATGCTCTGCGAAGTAATGATGATCTCAAAGAGAACATCGCCATCGTGGAGAGACGCAACAATCTGCTGCAGGCTGAACTGGATGAGCTGAGATCTCTGGTGGAACAGACTGAGAGAGGAAGGAAACTGGCTGAGCAGGAACTGCTGGACGTCAGCGAGAGAGTTCAGCTCCTGCATTCTCAGAACACCAGTCTGCTGAATCAGAAGAAGAAGCTGGAGGGAGATAATACTCAGCTTCAGACTGAGGTTGAGGAGGCAGTGCAGGAGTGCAGGAACGCTGAGGAAAAGGCCAAGAAGGCCATCACTGATGCTGCCATGATGGCTGAGGAGCTGAAGAAGGAGCAGGACACCAGTGCTCACCTGGAGAGGATGAAGAAGAACATGGAGCAGACCATCAAGGACCTGCAGCACCGTCTGGATGAAGCTGAGCAGATCGCCATGAAGGGAGGCAAGAAGCAGGTCCAGAAACTGGAAGCCAGGGTGAGAGAGCTGGAAAATGAGGTGGAGCTGGAACAGAGAAAGGCGAGCGAGTCTGTGAAAGGAGTGCGTAAATATGAGAGACGCATCAAGGAGCTCACCTACCAGACTGAGGAGGACCGTAAGAATCTGGCTCGTCTTCAAGACCTCGTGGATAAACTCCAGCTGAAGGTCAAGTCCTACAAGAGAGCGGCTGAGGAGGCGGAGGAGCAGGCCAATTCTAACCTGGGCAAGTTCCGTAAGATTCAGCATGAGCTGGATGAAGCAGAGGAGAGAGCTGATATTGCTGAATCTCAGGTCAACAAGCTGAGAGCCAAAAGCAGAGACACCAGATCTAAGAAAGGACATGATGAGGAATGAAGCTCTGCCATCTCTCAGAATATGTTGTGTCTCTTTGAATCCATTAGTTTCTAGATCAGTAGAATAAAGCAAATCTGCAGATTAAA

>Embrionic_myosin_heavy_chain_1_(Emb_myhc1)

ACTGAATGAGTCTGTTGTGCAGCTTTACCAGAAGTCTTCTGTCAAACTGCTGGCTAGTCTCTACCCACCTGTTGTTGAAGAAACCGGTGGCAAGAAGGGAGGAAAGAAGAAGGGTGGTTCCATGCAGACTGTGTCCTCCCAGTTCAGGGAGAATTTGGGCAAGCTTATGACCAACTTGAGGAGCACTCACCCTCACTTTGTGCGTTGTCTGATTCCCAATGAGTCCAAGACTCCAGGTCTGATGGAGAACTTCCTGGTTATCCACCAGCTCAGGTGTAATGGTGTGCTGGAGGGCATCAGAATCTGCAGAAAGGGCTTCCCCAGCAGAATCCTCTATGGTGACTTCAAGCAGAGATACAAGGTGCTGAATGCCAGTGTAATCCCAGAGGGGCAGTTTATTGACAACAAGAAGGCTTGTGAGAAACTCCTGGGATCCATAGATATCGATCATGACCAGTACAGATTTGGACACACAAAGGTGTTCTTCAAAGCTGGTCTTCTGGGTACTCTTGAGGAGATGCGTGATGAGAAACTGGCTGCTCTGGTCACAATGACTCAAGCTCTTTGCCGTGGTTATGTGATGAGGAGGGAGTTTGTGAAGATGATGGAGAGGAGGGAGTCCATTTACACCATCCAATACAACATCCGCTCATTCATGAATGTCAAACACTGGCCATGGATGAAGGTTTACTACAAGATTAAGCCTCTGCTGAAGAGTGCTGAGACTGAGAAGGAGCTGGCAACCATGAAAGAGGACTTTACAAAATGCAAAGAAAATCTTGCCAAGGCTGAAGCCAAAAAGAAGGAGCTTGAAGAGAAGATGGTGGCACTGCTGCAAGAGAAAAATGATCTGCAGCTGCAAGTGGCTTCTGAATCTGAGAATCTCTCAGATGCTGAGGAAAGGTGTGAGGGTCTGATCAAGAGCAAAATCCAGCTTGAAGCTAAACTCAAAGAGACAACTGAGAGACTGGAGGATGAAGAAGAAATCAATGCTGAACTGACAGCCAAGAAGAGAAAACTTGAGGATGAGTGCTCTGAGCTAAAGAAAGACATTGATGACCTGGAGCTGACCTTGGCTAAAGTGGAAAAGGAGAAACACGCCACTGAGAATAAGGTCAAGAACTTGACTGAGGAAATGGCATCTCAGGATGAGAGCATTGCCAAACTTACAAAAGAGAAGAAAGCTCTCCAAGAGGCACATCAGCAGACCCTGGATGATCTTCAGGCAGAGGAGGACAAAGTCAACACCCTGACCAAATCCAAGACAAAACTTGAGCAGCAAGTTGATGATCTTGAGGGCTCCCTTGAACAAGAGAAGAAGCTCCGCATGGATCTTGAGAGAGCAAAGAGAAAGCTTGAAGGAGACCTGAAATTAGCACAAGAGTCCATCATGGACCTGGAGAATGACAAGCAGCAATCTGACGAGAAACTTAAAAAGAAAGACTTTGAAACAAGCCAGTTGCTCAGCAAGATTGAAGATGAACAATCTCTGGGTGCTCAACTCCAGAAGAAGATCAAGGAGCTTCAGGCTCGCATTGAGGAACTGGAGGAAGAGATTGAAGCTGAGCGTGCTGCTCGTGCCAAGGTTGAGAAGCAGAGAGCTGATCTCTCCAGGGAACTTGAGGAGATCAGTGAGAGGCTTGAGGAGGCTGGAGGAGCCACTGCTGCTCAGATCGAGATGAATAAGAAGCGTGAAGCTGAATTCCAGAAGCTGCGTCGTGATCTTGAAGAGTCCACCCTCCAGCATGAAGCTACTGCTGCAGCCCTCCGTAAGAAGCAGGCAGACAGTGTGGCCGAGCTGGGAGAGCAAATCGACAACCTCCAGCGCGTCAAGCAGAAGCTTGAGAAAGAGAAGAGTGAATACAAAATGGAGATTGATGATCTCTCCAGCAACATGGAGGCTGTTGCCAAAGCAAAGGGTAATCTTGAGAAGATGTGCCGCACCCTTGAGGACCAACTTAGTGAAATTAAGTCCAAGAATGATGAGAACAACCGCCAGATAAATGACCTCAGTGCTCAAAGAGCAAGACTTCAAACTGAAAATGGTGAGTTTGGCCGTCAGCTGGAGGAGAAGGAGGCTCTGGTTTCTCAGCTCACCAGAGGCAAACAAGCTTTCACTCAGCAAATTGAGGAGCTTAAGAGGCAGATTGAAGAGGAGGTTAAGGCTAAGAATGCACTGGCCCATGCTGTACAATCAGCCCGTCATGACTGCGACCTGCTCCGTGAGCAGTTTGAGGAAGAGCAGGAGGCAAAGGCTGAGCTGCAGCGGGGAATGTCAAAGGCCAATAGTGAAGTTGCTCAGTGGAGAACCAAATATGAAACTGATGCCATCCAACGCACTGAGGAGCTTGAAGAGTCCAAGAAGAAGCTGGCTCAGCGCCTTCAAGATGCAGAGGAACAAATTGAGGCTGTGAACTCTAAATGTGCATCTCTGGAGAAGACCAAACAGAGACTCCAGGGTGAGGTGGAGGACCTCATGATTGACGTGGAGAGGGCCAACTCTTTGGCTGCCAATCTTGACAAGAAGCAGAGGAACTTTGACAAGGTCCTGGCAGAATGGAAGCAGAAATATGAAGAAGGTCAGGCAGAGCTGGAAGGAGCCCAGAAAGAGGCTCGTTCACTCAGCACTGAGCTGTTCAAGATGAAGAACTCCTATGAGGAGTCTCTGGATCAGCTGGAGACCCTCAAGAGAGAGAACAAGAATCTGCAGCAGGAGATTTCAGATCTGACAGAGCAGTTAGGTGAGACAGGTAAGAGCATCCATGAGCTGGAAAAGGCCAAAAAAGCAGTGGAGACTGAGAAGTCAGAGATTCAGACCGCCCTGGAGGAGGCTGAAGGCACTCTGGAGCACGAGGAGTCCAAGATTCTCCGTGTCCAGCTTGAGCTAAACCAGGTCAAGGGTGAGGTTGACAGGAAGCTTGCAGAGAAGGATGAGGAGATGGAGCAGATCAAGAGGAACAGCCAGAGAGTCATTGAATCCATGCAGAGCACTCTGGACTCTGAGGTCAGGAGCAGGAATGATGCCCTGAGAATCAAGAAGAAGATGGAGGGAGACCTTAATGAGATGGAGATTCAGCTGAGCCACGCCAATCGCCAGGCTGCTGAGGCCCAGAAACAGCTCAGGAACGTCCAAGGACAACTCAAGGATGCCCAACTGCACCTTGATGAAGCTGTCAGAGGACAGGAGGACATGAAGGAGCAGGTGGCCATGGTGGAGCGCAGAAACACTCTGATGCAAGCTGAGATTGAGGAGCTGAGAGCTGCTCTGGAGCAGACAGAGAGAGGCCGCAAAGTGGCTGAACAAGAGCTGGTGGACGCCAGTGAGCGTGTCGGACTGTTGCACTCTCAGAACACAAGTCTCCTGAACACCAAGAAGAAGCTTGAGGCTGACCTTGTTCAGATCCAGAGTGAAGTTGATGACACTATTCAGGAAGCCAGAAATGCAGAGGAGAAGGCCAAGAAGGCCATCACTGATGCTGCCATGATGGCTGAGGAGCTGAAGAAGGAGCAGGACACCAGTTCTCACCTGGAGAGGATGAAGAAGAACATGGAGGTGACTGTCAAAGACCTGCAGCACCGTCTGGATGAGGCTGAGAATCTGGCCATGAAGGGAGGAAAGAAACAACTCCAGAAACTGGAGAGTAGGGTCCGTGAGCTTGAGGCTGAAATTGAAGCAGAACAGAGACGTGGAACAGATGCTGTTAAGGGTGTCCGTAAATATGAGAGGAGAGTGAAAGAGCTGTCTTATCAGACTGAGGAGGATAAGAAGAACATCAACAGACTTCAGGATCTGGTTGATAAGCTGCAGTTGAAGGTCAAAGCCTACAAAAGACAGGCTGAGGAGGCGGAAGAGCAAGCCAACTCTCACATGTCCAAGTTGAGGAAGGTGCAGCATGAGCTGGAGGAGGCTGAGGAGCGTGCTGACATTGCTGAGTCTCAAGTCAACAAGCTCAGAGCCAAGAGCCGTGATGCTGGAAAGGCCAAAGAAGAGTGAAACCCTCAAACCTGTTGGAAGCCTCTACTGAAGTCATATAATATGATTGTTCTGTA

>Myosin, heavy polypeptide 1.1b_(Myhz1.1b)

GCACCCTGGAGCATGAAGAGTCCAAGATTCTCCGTGTGCAGCTGGAGCTGAACCAGGTGAAGAGTGAGATTGACAGGAAGCTGGCTGAGAAGGATGAGGAGATGGAACAGATCAAGAGGAACAGCCAAAGAGTGATCGACTCCATGCAGAGCACTCTGGACTCTGAGGTCAGGAGCAGAAATGATGCCCTGAGAGTCAAAAAGAAGATGGAGGGAGATCTGAATGAGATGGAGGTCCAGCTGAGTCATGCCAACCGCCAGGCTGCTGAGGCCCAGAAACAGCTCAGGAACGTCCAAGGACAACTCAAGGATGCCCAACTGCACCTTGATGAAGCTGTCAGAGGACAGGAGGACATGAAGGAGCAGGTGGCCATGGTGGAGCGCAGGAATGGCCTGATGCAAGCAGAGATTGAGGAGCTGAGAGCTGCACTGGAGCAAACAGAGAGAGGCCGCAAAGTGGCTGAGCAGGAGCTGGTGGATGCCAGCGAGCGTGTGGGACTGCTGCACTCACAAAATACGAGTCTTATTAACACCAAGAAGAAGCTTGAGACTGATCTGGTCCAGGTTCAAGGTGAGGTGGACGATGCAGTCCAGGAGGCCAGAAATGCAGAGGAGAAGGCCAAGAAGGCCATCACTGATGCTGCCATGATGGCTGAGGAGCTGAAGAAGGAGCAGGACACCAGTTCTCACCTGGAGAGGATGAAGAAGAACATGGAGGTGACTGTCAAAGACCTGCAGCACCGTCTGGATGAGGCTGAGAGTCTGGCCATGAAGGGTGGAAAGAAACAGCTCCAGAAACTGGAGTCCAGGGTGCATGAGTTGGAGGCTGAAGTTGAAGCTGAACAGAGACGTGGTGCAGACGCTGTGAAAGGAGTGCGCAAATATGAAAGGAGAGTGAAGGAGCTCACCTACCAGACTGAGGAAGACAAGAAGAATGTGGCTCGGCTGCAGGATCTGGTAGACAAGCTACAGCTGAAAGTGAAGGCATATAAACGCCAAGGCTGAAGAAGCTGAGGAGCAGGCCAACACTCACCTGTCCCAGGTACAGGAAGGTGCAGCATGAGCTGGAGGAGGCTCAGGAGCGCGCTGATATCGCCGAGTCCCAGGTCAACAAGCTGAGAGCCAAGAGCCGTGATGCTGGGAAGACTAAAGATGAAGAATGAAGACAAGAAACTACACCTACAAGCAAGCATATAATATGACTGACTTGTGCTGAGTTTTCCTGTGTCCATTAA

>Myosin, heavy polypeptide 1.1c(Myhz1.1c)

AAGTACAATCATGGGAGATGGTGAAATGGAGTGTTTCGGCCCGGCGGCCATTTACCTCCGGAAGCCAGAAAGAGAGCGAATTGAGGCTCAGAACACCCCCTTTGATGCCAAAACGGCATTCTTTGTGGTAGATGCAGATGAGATGTACCTGAAGGGTACCCTTGTTAGCAGAGAGGGTGGCAAAGCTACTGTCAAAACTCACAGTGGGAAAACTGTCACGGTAAAAGAAGATGAAATCTTCCCCATGAATCCTCCCAAGTTTGACAAAATTGAGGACATGGCCATGATGACCCACCTCAACGAACCCGCTGTGCTGTATAACCTCAAAGAGCGTTACGCAGCATGGATGATCTATACCTACTCTGGCTTGTTCTGCGTCACTGTCAATCCCTACAAGTGGCTCCCAGTGTACGACACAGTTGTTGTGGGTGGATACAGAGGCAAAAAGAGGATTGAAGCCCCACCTCACATCTTCTCCATCTCTGACAATGCCTATCAGTTCATGCTCACTGATCGTGAGAATCAGTCTATCCTGATTACTGGAGAATCTGGTGCAGGAAAGACTGTCAACACAAAACGTGTCATTCAGTACTTTGCAACTGTTGGTGCAATGTCTGGACCGAAGAAGCCAGAGCCAGTCCCTGGAAAAATGCAGGGATCACTGGAGGACCAGATTGTGGCAGCCAACCCTCTGCTGGAGGCTTATGGTAACGCCAAGACTATGAGGAATGACAACTCCTCCCGCTTTGGTAAATTCATCCGAATTCATTTTGGCACCACTGGAAAACTGGCATCAGCTGATATTGAAACTTATCTGCTGGAAAAGTCGAGAGTGACATTCCAGCTGTCGGCTGAGAGGAGTTATCACATCTTCTACCAGCTCATGACTGGACACAAGCCAGAGCTGCTCGAGGCCTTGCTCATCACCACCAACCCTTACGACTATCCAATGATAAGTCAGGGGGAAATCACTGTCAAGAGCATCAATGATGTGGAGGAGTTCATTGCTACAGATACTGCCATTGACATTCTGGGGTTCAATGCTGATGAGAAAGAGAAAATCAGCATCTACAAGCTGACAGGTGCTGTGATGCATCATGGGAACATGAAGTTCAAACAGAAGCAGAGAGAGGAGCAAGCTGAACCTGACGGCACTGAGGTGGCTGATAAAATCGCTTACCTCTTGGGCATCAACTCCGCTGACATGCTGAAAGCTCTGTGCTACCCCAGAGTGAAGGTTGGAAATGAGATGGTGACCAAAGGCCAGACAGTACCACAGGTGAACAACGCAGTCTCGGCTCTTTGCAAGTCTGTCTATGAGAAAATGTTCTTGTGGATGGTCGTCCGTATCAATGAGATGCTGGACACAAAGCAGCCTAGACAGTTCTTCATTGGTGTGCTGGACATTGCTGGATTTGAGATCTTTGATTTCAACAGCTTGGAGCAGCTTTGCATCAACTTCACAAATGAAAAACTGCAACAGTTCTTTAACCACCACATGTTTGTTCTGGAGCAAGAGGAGTACAAGAAAGAAGGCATTGATTGGGAGTTCATTGACTTTGGTATGGACTTGGCTGCCTGCATTGAGCTCATTGAGAAGCCAATGGGCATCTTCTCCATCCTTGAAGAGGAGTGCATGTTCCCCAAGGCAACAGACACAAGCTTCAAAAACAAGCTGCATGATCAGCATCTGGGCAAATGTGCAGCTTTCCAGAAGCCCAAGCCTGCCAAAGGTAAGGCCGAGGCCCACTTCTCTCTGGTGCACTACGCCGGCACTGTGGACTACAACATCGTCGGCTGGTTGGACAAGAACAAGGATCCATTGAACGACTCTGTCGTGCAACTTTACCAAAAGTCATCTATGAAAGTACTGGCCTTCCTGTATGCCGCTCATGGAGCTGCTGAAGCTGAGGGTGGCGGTGGAAAGAAAGGCAAGAAGAAGGGTGGTTCCTTCCAGACGGTGTCTGCAGTTTTTAGGGAGAACTTGGGTAAGCTGATGACTAACCTGAGGAGCACTCACCCTCACTTTGTGCGCTGCTTGATTCCTAATGAGTCCAAGACTCCAGGTCTGATGGAGAACTTCCTGGTTATCCACCAGCTCAGGTGTAATGGTGTGCTGGAGGGTATCAGAATCTGCAGGAAGGGTTTCCCCAGCAGAATTCTATATGGTGACTTCAAGCAGAGATACAAAGTATTAAATGCTAGTGTCATCCCTGAGGGACAGTTCATTGACAACAAAAAGGCTACAGAGAAACTCTTGGGCTCTATTGATGTTGACCACACCCAATACAAATTTGGACACACCAAAGGTGTTCTTCAAAGCTGGTCTGTTGGGTACTCTTGAGGAGATGAGAGATGAGAAACTAGCATCACTGGTTACCATGACTCAGGCTTTGTGCCGTGGATATGTCATGAGAAAGGAGTTTGTCAAAATGATGGAAAGGAGAGAATCAATTTATTCCATCCAATACAACATCCGCTCATTCATGAATGTCAAACATTGGCCATGGATGAAGCTCTACTTCAAGATCAAGCCTCTTCTGAAGAGTGCAGAGACTGAGAAAGAGATGGTAGCAATGAAGGAGAATTACGAAAAAATGAAGGAGGATCTGGCAAAGGCATTAGCTAAAAAGAAGGAGCTTGAGGAGAAAATGGTGTCACTAATTCAGGAGAAAAACGACCTTCAGCTGCAAGTAACAGCTGAATCTGAAAACCTCTCTGATGCTGAGGAGAGATGTGAAGGGCTCATCAAAAGCAAGATCCAGCTCGAGGCCAAACTCAAAGAAACAAACGAGAGACTGGAGGATGAGGAGGAAATCAATGCTGAACTGACTGCCAAGAAGAGAAAACTGGAGGACGAATGCTCCGAGCTGAAGAAAGACATCGATGACCTGGAGCTCACCTTGGCAAAAGTGGAGAAGGAGAAACATGCTACAGAAAATAAGGTGAAAAACCTGACAGAGGAGATGGCCTCTCAGGATGAGAGCATCGCCAAGCTGACCAAAGAGAAGAAAGCCCTCCAAGAGGCACACCAGCAGACTCTTGATGACCTTCAGGCAGAGGAAGACAAAGTCAACACTCTGACTAAAGCTAAGACAAAGCTTGAGCAGCAAGTGGACGATCTTGAGGGCTCACTGGAGCAAGAGAAGAAGCTCCGTATGGACCTTGAGAGAGCCAAGAGAAAGCTTGAGGGTGATCTGAAACTGGCCCAGGAGTCCATAATGGACCTGGAGAATGAAAAACAGCAATCAGATGAGAAGATCAAAAAGAAGGACTTTGAGATAAGTCAACTTCTCAGCAAGATTGAGGATGAACAGTCTTTGGGAGCACAGCTTCAGAAGAAGATCAAAGAACTTCAGGCCCGTATCGAGGAGCTGGAAGAGGAAATAGAGGCAGAGCGAGCTGCTCGTGCTAAAGTGGAGAAGCAGAGAGCTGATCTCTCCAGGGAACTTGAAGAGATCAGCGAGAGGCTTGAGGAAGCTGGTGGTGCTACTGCTGCTCAGATTGAGATGAACAAGAAGCGTGAAGCTGAATTCCAGAAGTTGCGTCGTGATCTGGAGGAGTCCACCTTGCAGCATGAAGCTACAGCTGCAGCTCTCCGAAAGAAGCAGGCAGACAGTGTGGCTGAACTCGGAGAACAGATCGACAACCTCCAGCGGGTCAAGCAGAAGCTGGAGAAGGAGAAGAGTGAATACAAGATGGAGATTGATGACCTGACAAGCAACATGGAGGCTGTGGCTAAAGCAAAGGCTAATTTAGAGAAGATGTGCCGAACCCTGGAAGACCAGCTGAGTGAAATCAAGACCAAGAGTGATGAAAATGTTCGTCAGCTGAATGACATGAATGCACAACGTGCAAGACTTCAGACTGAAAATGGTGAATTTAGCCGTCAACTGGAAGAGAAAGAAGCACTTGTTTCACAGCTAACTAGAGGAAAACAAGCTTATACACAGCAAATTGAGGAACTCAAAAGGCATATTGAGGAAGAAGTCAAGGTCAAGAATGCTCTGGCCCATGCGGTTCAGTCTGCCCGTCATGACTGCGACTTGCTCAGAGAGCAGTATGAGGAGGAGCAGGAGGCCAAAGCTGAACTCCAGCGGGGAATGTCTAAGGCCAACAGTGAGGTGGCCCAATGGAGAACCAAATATGAGACTGATGCCATTCAGCGGACCGAGGAGCTTGAGGAATCCAAGAAAAAGCTTGCCCAGCGTCTGCAGGATGCTGAAGAATCCATTGAAGCGGTGAACTCCAAGTGTGCCTCTCTGGAAAAGACCAAACAGAGACTGCAGGGTGAAGTAGAGGACCTCATGATTGATGTGGAGAGGGCAAATTCATTGGCTGCCAACCTTGACAAGAAGCAGAGAAACTTTGACAAGGTCCTAGCAGAGTGGAAACAGAAGTATGAGGAAAGCCAGGCTGAACTTGAAGGAGCTCAGAAAGAAGCTCGTTCTCTCAGCACTGAGCTTTTCAAAATGAAGAACTCCTATGAGGAAGCTCTTGACCACCTCGAGACCCTGAAGAGGGAGAACAAGAATCTGCAACAGGAGATTTCTGACCTCTCTGAGCAGCTTGGAGAGACTGGAAAGAGCATTCATGAGATAGAGAAAGCCAAGAAGACAGTGGAGTCCGAGAAATCAGAGATCCAGACCGCACTTGAAGAAGCTGAGGGCACCCTGGAGCATGAAGAGTCGAAGATTCTCCGTGTGCAGCTGGAGCTGAACCAGGTGAAGAGTGAGATTGACAGGAAGCTGGCTGAGAAGGATGAGGAGATGGAACAGATCAAGAGGAACAGCCAAAGAGTGATTGATTCCATGCAGAGCACTCTGGACTCTGAGGTTAGGAGCAGAAATGATGCCCTGAGAGTCAAAAAGAAGATGGAGGGAGATCTGAATGAGATGGAGGTCCAGCTGAGTCATGCCAACCGCCAGGCTGCTGAGGCCCAGAAACAGCTCAGGAACGTCCAAGGACAACTCAAGGATGCCCAACTGCACCTTGATGAAGCTGTCAGAGGACAGGAGGACATGAAGGAGCAGGTGGCCATGGTGGAGCGCAGGAATAACCTGATGCAAGCAGAAATTGAGGAGCTGAGAGCTGGCCTGGAGCAAACAGAGAGAGGACGCAAAGTGGCTGAGCAGGAGCTGGTGGATGCCAGCGAGCGTGTGGGACTGCTGCACTCACAAAATACAAGTCTTATTAACACCAAGAAGAAGCTTGAGGCTGATCTGGTCCAGGTTCAAGGAGAGGTGGATGATGCAGTCCAGGAGGCCAGAAATGCAGAGGAAAAGGCCAAGAAGGCCATCACTGATGCTGCCATGATGGCTGAGGAGCTGAAGAAGGAGCAGGACACCAGTGCTCACCTGGAGAGGATGAAGAAGAACCTGGAGGTGACTGTCAAAGACCTGCAGCACCGTCTTGATGAGGCTGAGAGTCTTGCCATGAAGGGTGGAAAGAAACAGCTCCAGAAACTGGAGTCCAGGGTGCGCGAGTTGGAGGCTGAAGTTGAAGCTGAACAGAGACGTGGAGCAGACGCTGTGAAAGGAGTGCGCAAATATGAAAGGAGAGTTAAGGAGCTCACCTACCAGACTGAGGAAGACAAGAAGAACGTGATCCGACTGCAGGATCTGGTAGACAAGCTGCAGCTGAAAGTGAAGGCCTACAAGCGCCAGGCTGAAGAAGCTGAGGAGCAGGCCAACACTCACCTGTCCAGGTACAGGAAGGTGCAGCATGAGCTGGAGGAGGCTCAGGAGCGCGCTGATATCGCTGAGTCCCAGGTCAACAAGCTGAGAGCCAAGAGCCGTGATGCTGGGAAGACTAAAGATGAAGAATGAAGAGAAGAAACTACACCTACAAGCAAGCATATAATATGACTGACTTGTGCTG

>Myosin, heavy chain b_(myhb)

CTGTGTGGCAAAACCATCACAGTGAAGGAGGATGACATTTTTCCAATGAATCCCCCCAAGTTCGATAAGATAGAGGACATGGCCATGATGACCCACCTCAATGAGCCTGCTGTGCTGTATAACCTTAAAGAGCGTTATGCAGCATGGATGATCTATACCTATTCCGGGTTGTTCTGTGTCACTGTGAACCCCTATAAGTGGCTCCCAGTGTATGATTCTGCAGTTGTGGCAGCCTACAGGGGCAAAAAAAGAATTGAAGCCCCACCTCACATCTTCTCCATCTCTGACAATGCCTATCAGTTCATGCTCACTGATCGTGAGAATCAGTCTATCCTGATTACTGGAGAATCTGGTGCAGGAAAGACTGTGAACACCAAACGTGTCATCCAGTACTTTGCGACAATCGCTGTATCATCTGGACAGAAGAAAGCAGAGCCCGTCCCTGGAAAAATGCAGGGGTCGCTGGAAGATCAAATCATTGCAGCCAACCCCTTGCTGGAAGCCTATGGAAATGCCAAGACTGTGAGGAATGACAACTCCTCCCGCTTTGGTAAATTCATCCGAATTCATTTTGGCACCACTGGAAAACTGGCATCAGCTGATATTGAAACTTATCTGCTGGAAAAGTCAAGAGTAACTTTCCAGTTGTCTGCTGAGAGGAGCTACCACATCTTCTACCAGCTTTGCACTGGCCATAAGCCAGAACTGCTGGAGGCTCTTCTAATCACCACTAACCCATTCGACTATCCCATGATCAGTCAGGGTGAGATCACAGTCAAGAGTATCAATGATGTGGAAGAGTTCATTGCAACTGATACAGCCATTGATATCCTGGGCTTTACTGCTGAGGAGAAAATGGGCATCTACAAGCTGACAGGAGCTGTGATGCATCATGGGAACATGAAGTTCAAACAGAAGCAGAGAGAGGAGCAAGCTGAACCTGATGGCACTGAGGTGGCTGATAAAATCGCTTACCTCTTGGGGCTCAACTCCGCTGACATGCTGAAAGCTCTGTGTTACCCCAGAGTGAAGGTCGGGAATGAATTTGTGACCAAAGGTCAAACTGTTCCTCAGGTGAATAATTCAACCATGGCTCTGTGCAAGTCAGTTTATGAGAAAATGTTCTTGTGGATGGTTGTCCGAATCAATGAGATGCTGGACACCAAACAGCCAAGGCAGTTCTTCATCGGAGTGCTTGATATTGCTGGGTTTGAAATCTTTGATTTCAACAGCCTGGAGCAGCTGTGCATTAATTTCACAAATGAGAAACTGCAACAGTTTTTCAACCACCACATGTTTGTGCTGGAGCAAGAGGAGTACAAGAAAGAAGGCATTGATTGGGAGTTCATTGATTTCGGTATGGACTTGGCTGCCTGCATTGAGCTTATTGAAAAGCCAATGGGTATTTTCTCCATCCTTGAGGAGGAGTGTATGTTCCCTAAGGCAACAGACACAACCTTTAAGAACAAGCTGCATGACCAGCATCTTGGTAAAAGTGCATGCTTCCAGAAGCCCAAACCTACCAAAGGCAAGGCCGAGGCCCACTTCTCCTTGGTGCACTATGCTGGAACTGTGGATTACAACATCGTAGGCTGGCTGGATAAGAACAAGGACCCTCTGAATGACTCAGTGGTTCAGCTGTACCAGAAGTCCTCAATGAAACTGCTGGCTCACCTGTATGCTGCTCATGCTTCTGCTGAAGCTGACACTGGTGGCAAAAAGGGTGGGAAAAAGAAGGGCGGCTCCTTCCAAACTGTGTCTGCTCTGTTCCGGGAGAACTTGGGCAAACTGATGACCAACCTGAGAAGCACTCATCCTCATTTTGTCCGTTGCTTGATTCCAAATGAGTCAAAGACTCCAGGTCTTATGGAGAACTTTCTGGTCATCCATCAGTTGCGCTGTAACGGTGTACTGGAGGGAATCAGAATTTGCAGAAAAGGTTTCCCAAGCAGAATCCTCTATGGTGACTTCAAGCAAAGATACAAAGTATTGAATGCTAGTGTCATCCCTGAGGGTCAGTTCATTGACAATAAGAAGGCTTCAGAGAAGCTTCTTGGCTCCATTGATGTGGATCATACCCAGTACAAGTTTGGACACACCAAGGTGTTCTTCAAAGCTGGTCTTCTGGGTCTTCTTGAGGAGATGCGAGATGAAAAACTTGTTATCCTTGTGACCATGACTCAAGCACTTTGCAGAGGCTATGTTATGAGGAAGGAGTTTGTCAAAATGATGGAGAGAAGAGAGTCCATCTTCACCATCCAGTACAACATCCGCTCATTCATGAATGTGAAACACTGGCCATGGATGAAGCTGTATTTTAAGATCAAGCCTCTGCTCAAGAGTGCAGAGACTGAGAAGGAAATGGCAGCCATGAAAGAGAATTTTGAAAAAATGAAGGAGGATCTAGCAAAGGCACTTGCCAAGAAGAAGGAGCTGGAGGAGAAGATGGTTTCCCTGCTGCAGGAGAAAAATGACCTGCAGTTGCAAGTGGCAGCTGAAACAGAGAACCTTTCTGATGCTGAGGAGAGATGCGAAGGACTAATCAAAAGCAAGATCCAGCTGGAGGCGAAACTCAAAGAGACAAATGAAAGACTGGAGGATGAGGAAGAAATCAATGCTGAACTGACAGCCAAGAAGAGAAAACTAGAGGATGAGTGCTCTGAGCTGAAGAAAGACATTGATGATTTAGAGCTTACCTTAGCCAAAGTGGAGAAGGAGAAACATGCCACCGAGAACAAGGTTAAAAACCTTACTGAAGAATTGACATCTCAAGATGAGGTTATTGCTAAGCTGACTAAGGAGAAGAAAGCCCTCCAAGAGGCACATCAGCAGACTCTTGATGATCTGCAGGCAGAGGAGGACAAAGTCAATACTCTGACAAAAGCCAAAGCAAAGCTTGAGCAGCAAGTCGATGACCTGGAAGGTTCTCTGGAGCAGGAGAAGAAACTCCGTATGGACCTGGAGAGGGCCAAGAGAAAGCTTGAGGGTGACCTGAAACTAGCTCAAGAATCCATAATGGACCTGGAGAATGACAAGCAACAATCAGACGAGAAGATAAAGAAGAAGGACTTTGAATCAAGTCAATTGCTGAGCAGGATTGAAGACGAGCAGTCTCTTGGTATTCAGCTCCAGAAGAAGATTAAAGAGCTTCAGGCTCGTATTGAGGAGCTGGAGGAAGAAATTGAGGCAGAACGCGCTGCTCGTGCTAAGGTCGAGAAGCAGAGGTCTGATCTCTCCAGGGAACTTGAGGAGATCAGCGAGAGGCTTGAAGAAGCAGGAGGTGCCACTGCTGCACAGATTGAGATGAATAAAAAACGTGAGGCTGAGTTCCAGAAACTACGCCGTGATCTGGAGGAGTCCACCTTGCAGCACGAAGCGACGGCTGCAGCACTCCGCAAGAAGCAGGCTGACACTGTGGCAGAGCTGGGAGAACAGATTGACAACCTCCAGCGTGTGAAACAGAAGCTGGAAAAAGAGAAGAGTGAGTTTAAAATGGAGATTGATGACTTGTCAAGCAGTATGGAGGCTGTTGCCAAATCAAAGACAAATCTTGAAAAGATGTGCCGTACACTTGAAGATCAGTTGAGTGAGTTTAAGACAAAGCATGATGAACATGTACGCCACATCAATGATATAGGTGCTCAAAAGGCAAGACTTCAGACTGAAAATGGTGAAATGGGACGTCAACTAGAAGAAAAAGAGGCTTTAGTTTCTCAGCTGACTCGAAGCAAACAGGCTTTCACTCAGCAGATTGAAGAGCTGAAGAGGCAAATTGAGGAAGAAGTCAAGGCCAAAAATGCCTTGGCTCATGGTGTCCAGTCAGCACGACATGACTGTGATCTGCTCAGAGAGCAGTACGAGGAGGAACAGGAAGCCAAATCTGAGCTCCAGCGTGGCATGTCAAAGGCCAATAGTGAAGTAGCTCAATGGAGATCCAAATATGAGACCGATGCCATTCAGCGTACTGAAGAGCTTGAGGAAGCAAAGAAAAAGCTTGCTCAGCGTTTACAGGATGCTGAAGAGTCCATTGAGGCTGTGAATGCCAAATGTGCCTCCTTGGAAAAGACTAAACAGCGACTGCAAAATGAAGTCGAGGATCTCATGATTGATGTGGAGAGAGCAAATGCTTTAGCTGCCAACCTTGACAAGAAACAAAGGAACTTTGATAAGGTCTTAGCAGAGTGGAAGCAGAAGTATGAGGAAAGCCAGGCAGAGTTGGAAGGAGCCCAGAAGGAGGCTCGTTCTCTTAGTACAGAACTTTTCAAAATGAAAAATTCTTATGAGGAGGCTTTGGACCACCTGGAGACTCTGAAAAGGGAAAACAAGAACCTTCAACAGGAGATCTCAGATTTGACTGAGCAGCTTGGTGAAACTGGAAAGACTATTCATGAGCTGGAAAAAGGAAAGAAAACTGCTGAAATGGAAAAATCTGAAATCCAAGCCGCACTTGAAGAAGCTGAGGCAACCCTAGAGCATGAAGAATCAAAGATTCTTCGCGTTCAGCTTGAGTTGAACCAAGTGAAAGGGGAGATTGATCGGAAATTGGCTGAGAAGGATGAGGAGATTGAACAGATCAAGCGAAACAGCCAGAGAATCATTGATTCCATGCAGACCACCCTGGATGCTGAAGTCAGAAGCAGAAATGATGCTCTGAGAATTAAGAAGAAGATGGAAGGAGACCTCAATGAGATGGAGATTCAGTTGAGCCATGCAAATCGCCAGGCTGCTGAAGCTCAGAAACAGCTCAGGAACGTCCAGGGACAACTGAAGGATGCTCAACTCCACCTTGATGAAGCTTTAAGAGCACAAGAGGACATGAAAGAGCAGGTTGCTATGGTTGAACGCAGGAACAACCTGATGCAGGCTGAGATTGAGGAGCTGAGATCTGCTCTGGAGCAGACAGAGAGAGCTCGCAAAGTAGCTGAGCAGGAACTGGTGGATGCCAGTGAGAGAGTGACTCTCCTACACTCCCAAAATACCAGTCTTATTAACACCAAGAAGAAATTGGATGCAGATCTTGTTCAAATTCAAGGTGAGATGGAAGATGTAGTCCAGGAAGCACGCAATGCTGAAGAGAAGGCAAAGAAAGCAATAACTGATGCTGCCATGATGGCTGAGGAGCTGAAGAAGGAGCAGGACACCAGTGCTCACCTGGAGAGGATGAAGAAGAACCTGGAGGTGACTGTCAAAGACCTGCAGCACCGTCTGGATGAGGCTGAGAGTTTGGCCATGAAGGGAGGAAAGAAGCAGCTCCAGAAACTGGA

>Myosin, heavy polypeptide 1.1a_(Myhz1.1a)

acaatcatgggagatggtgaaatggagtgtttcggcccggcggccatttacctccggaagccagaaagagagcgactcgaggctcagaccatcccctttgatgccaaaacagcattcttcgtgacagatccagctgagatgtacttgaaaggtactcttcttagtatagaggctggcaaagctactgtcaaaactgactgtgggaaaactgtcaccgtaaaagaagatcaaatcttcccaaggaatcctcccaaatttgacaaaattgaggacatggccatgatgacccacctcaatgagcctgctgtgctgtataacctcaaagagcgttacgcagcatggatgatctatacctactctggtttgttctgtgccactgtcaatccatacaaatggctcccagtgtacgacgcagttgttgttgctggatacagaggcaaaaagaggattgaagccccacctcacatcttctccatctctgacaacgcctaccagttcatgctcactgatcgtgagaaccagtctgtcctgattactggagaatctggtgcaggaaagactgtgaacacaaaacgtgtcattcagtactttgcaactgttgcgatggctggtccaaagaagacagaggctgtccctggaaaaatgcagggatcactggaggaccagatcattgcagccaaccctctgctggaggcttatggtaatgccaagactataaggaacgacaactcctctcgttttttgggaaattcatcaggattcacttttcgggcactggtaaactggccaaagcagatattgaaacttatctgctagaaaagtcaagggtaacattccagctgtctgctgagaggagttaccacatcttctaccagctcatgactggacacaagccagagctgctcgaggccctgctcatcaccaccaacccttacgactatccaatgataagccagggtgaaatcactgtcaagagcatcgatgatgtggaggagttcattgccacagatactgccattgacattctgggcttcactgctgatgagaagataagcatctacaagctgacaggtgcggtgatgcatcatggggccatgaagttcaaacagaagcagagagaggagcaggccgaacctgacggcaatgaggcggctgataaaatcgcctacctcttgggcatcaactccgctgacatgctgaaagctctgtgtttccccagagtgaaggttggaaatgagatggtgaccaaaggccagacagtaccacaggtgaacaatgcagtctctgcactctgcaagtctgtctatgagaaaatgttcttgtggatggtcgtccgtatcaatgagatgttgaacacgacgaatcctagagagtactacatcggtgtgctggacatcgctggatttgagatctttgatttcaacagcttggagcagctttgcattaacttcacaaatgagaaactgcaacagttcttcaaccacaccatgtttgttctggagcaagaggagtacaagaaagaaggcattgaatgggcattcattgactttggtatggacttggctacctgcattgagctcattgagaagccaatgggcatcttctccattcttgaagaggagtgcatgttccccaaggcaacagactcaagcttcaaaaacaagctgcatgatcagcatctgggcaaatgttcagctttcgagaagcccaagcctgggaaaggtaaagccgaggcccacttctctctggtgcactacgccggcactgtggactacaacatcgtcggctggctggacaagaacaaggatccattgaacgactctgtcgtgcaactttaccaaaagtcagcactcaaagtgctggccttgctgtatctcgctgtgccagaagcagagggaggtggaaagaaagcaggcaagaagaagggtggttccttccagacggtgtctgcagttttcagggagaacttgggtaagctgatgactaacctgaggagcactcaccctcactttgtgcgctgcttgattcctaatgagtccaagactccaggtctgatggagaacttcctggttatccaccagctcaggtgtaatggtgtgctggagggcatcagaatctgcacgaagggtttccccagcagaatccactacggtgacttcaagcagaggtacaaagtattaaatgctgctgtcatccctgagggacagttcattgacaacaaaaaagctacagagaaactcttgggctctattgatattgaccacacccaatacaagtttggacacaccaaggtgttctttaaagctggtctgttgggtactcttgaggagatgagagatgaaaaactatcaagtctggttaccatgactcaggctttggcccgaggatatgtaatgaggaaggagtatgtcaaaatgacggagaggagggaggcaatttattccatccaatacaacatccgctcattcatgaatgtcaaacattggccatggatgaaggtgtacttcaagatcaagcctcttctgaagactgcagagagtgaaaaagaaatggcatcaatgaaggagaactttgataaaatgaaagaagatttaacaaggcattagctaaaaagaaggagcttgaggagaaaatggtgtcacttgttcaggagaaaaacgatcttctactgcaagtaacttctggaatctgaaaacctctctgatgctgaggagagatgtgaagggctcatcaaaagcaagatccaacttgagggcaaactcaaagagacaacagagagactagaagatgaggaggaaatcaatgctgaactgactgccaagaagaggaaactggaggatgaatgctctgagctgaaaaaagacattgatgacctggagctcaccttggcaaaagtggagaaggagaaacatgcaacagagaataaagtgaaaaacctgacagaggagatggcctctcaagacgagagcattgccaagctgaccaaagagaagaaagccctccaagaggcacaccagcagactcttgatgaccttcaggcagaggaagacaaagtcaacactctgactaaagctaagacaaagcttgagcagcaagtggacgatcttgagggctcattggagcaagagaagaaacttcgtatggaccttgagagagtcaagagaaagcttgagggtgatctgaaactggcccaggagtccataatggacctggagaatgaaaaacagcaatcagatgagaagatcaaaaagaaggattttgagataagtcagtttctcagcaagattgaggatgaacagtctttgggagcacagcttcagaagaagatcaaagaacttcaggcccgtatcgaggagctggaagaggaaattgaggcagagcgatctgctcgtgccaaagtagagaagcagagagccgatctttctagggaacttgaagagatcagcgagaggcttgaggaagctggtggtgctactgctgctcagattgagatgaacaagaagcgtgaagctgaattccagaagatgcgtcgtgatctggaggagtccaccttgcaacatgaagctacagctgcagccctccgaaagaagcaggcagacagtgtggctgaactcggagaacagatcgacaacctccagcgggtcaagcagaagctggagaaggagaagagtgaatacaagatggagattgatgatttgtcaagcaacatggaggctgtggctaaagcaaagggtaatttagagaagatgtgccgcacccttgaagaccagctgagtgaaatcaaggccaaaaatgatgaaaatagtcgccagttgaacgacatgagtgcacaacgagcaagacttcagactgaaaatggcgaatttagccgtcagctggaagagaaagaagcacttgtttcacagctaactagaggaaaacaggcttatacacagcaaattgaggaactcaaaaggcatattgaggaagaagtcaaggccaagaacgctctggcccacgcggttcagtctgcacgccatgactgtgatttgctcagagagcagtacgaggaggagcaggaggccaaagctgaactccagcggggaatgtctaaggccaacagtgaggtggcccagtggagagccaaatatgagacggatgccattcaacgcactgaagagcttgaggaatccaag

>Myosin_heavy_chain_11_(Myh11)

cacaggagttgctggctgaagagacgagacaaaaactccagttctccaccaaactacgtcaaatggaggatgaccgtaatgcccttcaggagcagcttgaggaagaatcggaagcaaagaggaatgtggagaggcaggtttcgacactgaacattcagctcgctgactttaagaagaagctggatgaggtgtcaggtaatgttgaacttttagaagaaggcaagaagcggctgcagagagacctggaggctgccaatactcagtttgaggagaaggcagcagctttcgacaagcttgagaagaccaagaacagactgcaacaggagcttgaggacacgctgatggatttggacaaccagagacagctggtgtccaaccttgagaagaaacagaagaagtttgatcagatgcttgctgaagagaagagcatctctaataagtatgcagatgagcgagatcgcgcagaggccgaggccagagagaaagagaccaaagctctttctttagccaaagctctcgaagaagcccaagaatcccgtgaggagctcgaaagagccaataaagcccttcgtgctgaaatggaggacctggtcagctccaaagatgatgtgggcaagagcgtacatgagcttgagaagtccaaacgtggcctggaggctcaggtagaagagatgaagactcagttggaagagctggaggatgaattacaggctgcagaagatgccaaactgcgcctggaagtcaacatgcaggctctgaaggcccagtttgagcgagatctccagggcagagatgagcaaggggaggagaagaagaggcagttgatcaaacaggtgcgtgagctggaaactgaactcgaagatgagcgtaagcaaagaactctaatagctgcttccaagaaaaaaatggagggagacataaaggaccttgagggccaagttgagacatctaacaagggacgagaagaggcgatcaagcagctccgcaagctccaggctcaaatgaaagacttccaaagagagcttgatgatgctcgagctgccagggaggaagttttatccagtgccaaggagagtgagaggaaggccaagactttggaagctgaacttctgcagatgcatgaggacttggctgcagctgagagggctaaaaagcaagctgaagctgagagagatgaactggctgacgagttggccagcaatgcatctggaaaatctgcactggctgatgagaagagacggttggaagcgaagattcaacagctggaggaagagctggaggaggagcagggcaatatggagatgcttaatgacaagctgaggaagagtgcccagcaggttgaccaacttaccaacgagctccaggctgaacgcaccacgtcccaaaagaacgagagcgccagacagttgatggagaggcagaataaagagctgaaagctaaactccaggagatggagagccaagtcaaatccaaatttaagtcctccatcactgctcttgaggccaaagtggcacaattagaggaacaacttgatcaggagagcagagaaaagcagaacacagcaaagacggttcgccagaaggataagaaactgaaggagttgatgacccaagtggaagatgaacgaaaacaagcagaacaatataaagaccaggcagataaggcgaacacgcgtgtcaaacagctgaagaggcagttggaggagagtgaggaggagtcccagcgaatcactgcggcccgcaggaagcttcagagagagctggacgaggccacagagaccaatgatgccatgagtcgtgaggtctcttccctcaagagcaaactcaga

>Myosin_heavy_chain_9b_(Myh9b)

ACCAGAGCAGCTCGGTCCTGTCACATGGTGAGCTGGAGAAGCAGCTGCTGCAGGCCAATCCCATCCTCGAAGCCTTCGGAAATGCCAAAACAGTCAAGAACGACAACTCTTCTCGATTCGGAAAGTTCATCAGAATCAACTTTGATGTTAACGGTTACATCGTCGGAGCCAATATTGAGACCTACCTGTTGGAAAAGTCCCGTGCAATCCGTCAAGCTAAAGAAGAGAGAACCTTCCACATGTTCTACTACATGCTCAGCGGTGTAGGAGACAAACTGCGCTCTGAACTCTGTCTGGAGAGCTACAGCAAGTATCGGTTCCTGTCCAACGGGAACGTGACAATCCCGGGACAGCAGGACCGAGACATGTACTTGGAAACTGTGGAGGCCATGAGGATCATGGGCTTCTCTGAGGAAGAACATATCGGTCTGTTGAGGGTAATTTCATCTGTGCTACAGCTGGGTAATATGTCCTTTAAGAAGGAACGTCACTCGGACCAGGCCTCCATGCCTGATGACACAGCTGCCCAGAAGGTGAGCCATCTGATGGGCATGAACGTGACTGATTTCACACGCGCCATCCTCTCGCCCCGGATCAAGGTGGGCCGTGACTATGTGCAGAAGGCCCAGACGCAGGAGCAGGCTGG

>Myosin_heavy_chain_a_(Myha)

ctccaagtgtgcctctctggaaaagaccaaacagagactgcagggtgaagtagaggacctcatgattgatgtggaaagagcaaattcattggctgccaaccttgacaagaagcagagaaactttgacaaagtcctagcagagtggaaacagaagtatgaggaaagccaggctgaactagaaggtgctcagaaagaagctcgttctctcagcactgagcttttcaaaatgaagaactcctatgaggaagctcttgaccacctcgagaccctgaagagggagaacaagaatctgcaacaggagatttctgacctctctgagcagcttggagagactggaaagagcattcatgagatagagaaagccaagaagacagtggagtctgagaaatcagagatccagactgcacttgaagaagctgag

>Myosin_light_chain_2_(Myl2)

GGCCTTCACTTCTTGAGCTTCTTAGACTTCACACATACCGTCTCAGCATGGCACCCAAGA

AGGCCAAGAGGAGGGCAGGAGGAGGAGAGGGTTCCTCCAACGTCTTCTCCATGTTTGAGC

AGAGCCAGATTCAGGAGTACAAAGAGGCTTTCACAATCATTGACCAGAACAGAGACGGTA

TCATCAGCAAAGACGACCTTAGGGACGTGTTGGCCTCAATGGGCCAGCTGAACGTGAAGA

ATGAGGAGTTGGAGGCCATGATCAAGGAAGCCAGCGGCCCAATCAACTTCACCGTTTTCC

TCACCATGTTCGGAGAGAAGCTGAAGGGTGCTGATCCCGAAGACGTCATTGTGTCTGCCT

TCAAGGTGCTGGACCCAGAGGGCACCGGCTTCATCAAGAAGCAATTCCTTGAGGAGCTTC

TGACCACTCAGTGCGATAGGTTCTCTGCAGAGGAGATGAAGAATCTGTGGGCCGCCTTCC

CCCCAGATGTTGCTGGCAATGTTGACTACAAGAACATCTGCTACGTCATCACACACGGAG

AGGAGAAGGAGGAGTAAACGGTCGTGGAGTCAAGACAGAAAATGAAGAGATGAACGTGCA

TCCCTCACTGCTTTACTCTCCCAGTCTGTTCTCTGTCCTCCTTCTCTTACTTTGTGTTTC

TTCCTCCCTTTCTTCCTTTCCATCCTCTTTGTTACTCTCAAGCACTTACTCTCTCCATCT

CTCCAAAGACTTGTCTCGCTGAGGCTGAATTGGGAGGGCGGAGAGGCTCATGACCACAGT

GTCCGTCAAGTGGGGATATGGGATTGTTTTCAATAAAATGAACATCAATACTGTATCTCT

CACATGCTCTCTCTTTCTCTCTGTTTCTCACTCACTACCCATGACCACCTCTC

>Myosin_light_chain_3_(Myl3)

CCGGCTTCTTGACTTCTGCCTGTCCGCTCCTGCACCCCAAAACTCCATCATGGCTGGAGAATTCTCTGCTGACCAGATTGAGGACTTCAAAGAGGCCTTTGGTCTCTTCGACAGAGTTGGTGATAACAAGGTTGCCTACAACCAGGTTGCTGACATCATGCGTGCCCTGGGACAGAACCCCACCAACAAGGACGTGAAGAAAATCTTGGGTGACCCATCTGCTGACGATATGGCCAACAAAAGAATTGACTTTGATGCTTTCCTGCCAATGCTGAAGACTGTTGATGCCGTCCAGAAGGGTACCTATGATGACTACGTTGAGGGTCTGCGCGTCTTCGACAAAGAGGGCAACGGCACAGTGATGGGCGCTGAGCTGCGCATTGTGCTCTCAACACTGGGTGAGAAGATGACTGAGCCCGAGATCGACTCTCTCATGCAGGGACAGGAGGATGAGAACGGCAGTGTCCACTATGAGGATTTCGTCAAGCACATCATGTCCGTGTAAGAGGCCGTCGGTTGAGAGAGTGGTGAAGAAGGCTGAACTCTATCTGCAGACCCCATGGTGTCAGGACATCCATTCTGTTTTGAAGACCAATCAATAAAAAGGACTATGGGATGCCACTTCTAAACCATTCTGTTCGTTTTCCTTTATTTGTTTTCTCCCCTCTCGCCCACCTTTTTGAAGTTACATCATCCACTCATGCAGTTGACCCTCCTTGCCTTCTTGTTCCGCCCGGGGCGGCCATCCACAGTAGGATGGAGACCGGGGAAGAAGGAAAAGGCCACTCATGAAGTGAGGGTTGGTTTCTTCTTTCTCCACCCATCCAGGGTTACAACAGCACCGCTAGCTGTGTGTTAAAGGCACATGTCATTGCTGTGGCCAGGCTGCCAATTTTTCCCCATCCCAATTTTCACACAGTCATTTCACAATAAACTTTTTTCCGGCTTCTTGACTTCTGCCTGTCCGCTCCTGCACCCCAAA

>Myosin_light_chain_9b_(Myl9b)

CCTGAAACTCCGTCATCATGTCCAGCAAGCGAGCAAAGGGAAAGACCACTAAGAAGAGGCCATAGAGAGCCACTTCAAACGTGTTTGCCATGTTTGACCAGTCACAGATCCAGGAGTTCAAGGAGGCCTTCAACATGATCGACCAGAACCGAGACGGATTCATCGATAAAGAGGATCTCCATGACATGCTGGCCTCTTTGGGTAAGAACCCGTCTGAAGATTACCTGGATGGGATGATGAGTGAAGCTCCGGGGCCCATCAATTTCACCATGTTCCTCACTATGTTTGGAGAACGGCTGAACGGAACCGACCCGGAGGACGTCATCAGGAACGCTTTCGCCTGCTTCGACGAGGAAGGATCCGGTTTCATTCATGAAGACCACCTGCGAGAGCTGCTGACCACCATGGGAGATCGTTTCACAGATGAAGATGTGGACGAACTCTTCAGAGAGGCTCCCATTGACAAAAAGGGCAACTTTAACTACGGCGAATTCACCCGGATTCTCAAACACGGGGCCAAAGATAAAGATGACATTTAGATGATCAGAGAGCGTCAGCTGCATGCATGTGCACAGACACATTACAAACACA

>Myosin_light_chain_9a_(Myl9a)

AGAAACAAACCCTGCGGTCAGAATGTCTGCAGCCAAGCGAGCCAAAGGAAAGACCACGAAGAAGCGCCCGCAGAGGGCCACGTCCAACGTCTTCGCCATGTTCGACCAATCACAGATCCAGGAGTTCAAAGAGGCCTTCAACATGATCGATCAGAACAGAGACGGATTCATCGATAAAGAGGATCTGCACGACATGCTGGCCTCTCTGGGGAAGAACCCGTCTGACGAGTACCTGGAGGGAATGATGAGCGAAGCTCCTGGTCCCATCAACTTCACCATGTTCCTCACCATGTTTGGAGAACGTCTGAACGG

>Myosin_light_chain_Myl12.1a_(Myl12.1a)

CCAAAAAGCGCCCCCAGCGGGCCACGTCGAACGTGTTTGCCATGTTCGACCAGTCCCAGATCCAGGAGTTCAAAGAAGCCTTCAACATGATCGACCAGAACCGCGACGGCTTCATCGATAAGGAGGATCTTCATGACATGCTCGCTTCTCTCGGGAAGAACCCAAAAGAAGACTATTTGGAGGCGATGATGACCGAAGCCCCCGGGCCCATAAACTTCACCATGTTCCTCACCATGTTCGGAGAGAAGCTGAACGGCACAGACCCCGAGGAAGTCATTCGTAATGCGTTCGCCTGCTTTGACGAGGAAGGAACAGGCTTCGTTCAGGAGGACTATCTGAGAGAGCTGCTGACCACTATGGGAGATCGATTCACAGACGAAGAGGTCGACGAGCTCTTCAGAGAAGCACCGATCGATAAAAAGAGCAACTTCAACTACGTGGAGTTCACCCGTATTCTTAAACACGGAGCCAAAGATAAAGACGATTAGAAAA

>Myosin_light_chain_Myl12.1b_(Myl12.1b)

TTTTCTAATCGTCTTTGTCTTTGGCTCCGTGTTTAAGAATACGGGTGAACTCCACGTAGTTGAAGTTGCTCTTTTTATCGATCGGTGCTTCTCTGAAGAGCTCGTCGACCTCTTCGTCTGTGAATCGATCTCCCATAGTGGTCAGCAGCTCCCTCAGATAGTCCTCCGGAACGAAGCCTGTCCCTTCCTCGTCAAAGCAGGCGAACGCATTACGAATGACTTCCTCAGGGTCTGTGCCGTTCAGCTTCTCTCCGAACATGGTGAGGAACATGGTGAAGTTTATGGGCCCAGGGGCTTCGGTCATCATCGCCTCCAAATAGTCTTCTTTTGGGTTCTTCCCGAGAGAAGCGAGCATGTCATGAAGATCCTCCTTATCGATGAAGCCGTCGCGGTTCTGGTCGATCATGTTGAAGGCTT

>Myosin_light_chain_Myl1b_(Myl1b)

ccaagaaagcagagcctgcacctgcccccgctcctgcgcccgcacccgaggccgcacctaaacccgcagcagtagatctgtccggtgtgaaggtcgatttcaaccaggaccagttggaagattacagggaagccttcggacttttcgacagagttggggacagcaaagtagcctacaaccagattgcagatatcatgcgtgcgctgggacagaacccaaccaacaaagaggttacaaagatcctgggcaaccctaccgccgatgacatggcaaacaagagagtcgactttgagggtttcctgcccatgctgcagtttgtggtcaacagcccaaacaaggcaacatacgatgactacgttgagggtctgcgtgtattcgataaggagggcaacggaacagtaatgggtgccgagttgcgtattgtcttgtcaacactgggtgagaaaatgaatgaaactgagatcgatgccctcatgcaaggccaggaggatgaaaatggctgtgtgaactatgaggctttcgtcaaacacatcatgtctgtgtaagaagtctgagttgtgaggaaactgaagcgtttctccagattccatgatgtcaggacatccacacagtatttccaaaaccaactcagaaatggatgaaagggacacgggatgttactcattaacaattattttgttatttaagtttcctattttttttccacttgactcttttgtccattcttggatgccattcatattttttccagcaaattttcttggtgttgttcatgccgggacaagcctctccgtaacggaatggttgtgaggggtgggggtgtaggggggcatcatagttacacaaaaatacaaaccaccccttgacccctcactgccaggatcacaaaatcaaaggtgctaaatattctgctattaatagactggctgcaaaacctccttcccctttccccagagtcttaatttattttttgcctctgtcaattctcataataaactttccacaaagt

>Myosin_light_chain_Myl1a_(Myl1a)

tgaaggtcgatttcaaccaggaccagatggaagattacagggaggccttcggacttttcgacagagttggtgacaacaaagtagcctacaaccagattgcagatatcatgcgtgcactgggacagaacccaaccaacaaagaggttacaaagatcctgggcaaccctacagctgatgatatggcaaacaagagagtcgactttgagactttcctgcccatgctgcagtttgtagtcaacagcccaaacaaggccacatacgaggactacgttgagggtctgcgtgtattcgataaggagggcaacggaacagtaatgggtgctgagctgcgtatcgtcttgtcaacactgggtgagaaaatgactgaaattgaaatcgatgctctcatgcaaggccaggaggatgaaaatggctgtgtgaactatgaggctttcgtcaaacacatcatgtctgtgtaagaagtcggagttgtgaggaaactgaagcgtttctcca

>Myosin_light_chain_Myl12.2a_(Myl12.2a)

AATAATTCCAGAATTTATTTCCCACATTTACATACCAGAACAGCATTCATTCACTTTGAGGTTTACCACAGATTACATGTCTATATATTTCATAGTTATGTACAAGCCTACATAGACTGTTTAAGAGTACAGTCAAATGACTTTGCCTTGCTGATTAATGTATCCCTTTGCACGATCCCAAGAATGTGGGGAAAAGAAGAAAGTTAAATATGTTGTAGTGTTATAGTAGAGTTTTCATTAGTTTCAGTAGTGCTGGGCCTTGTCCTAATCATCCTTATCCTTAGCACCATGTTTCAGGATGCGAGTGAATTCTACGTAGTTGAAGTTCCCTTTCTTGTCAATGGGGGCCTCTCTGAACAACTCGTCTACTTCTTCATCTGTGAACCTGTCTCCCATAGTGGTCAACAGCTCCCTTAGGTAGTCCTCCTGAATAAAACCCGTCCCCTCCTCATCAAAACAAGCAAATGCATTTTTGATAACATCTTCAGGATCTGTGCCATTAAGTTTCTCTCCAAACATTGTGAGGAACATGGTGAAGTTAATGGGACCAGGGGCTTCATTCATCATTGCCTCCAGGTATTCGTCTGTGGGGTTCTTACCTAATGAGGCCAGCATGTCATGCAGATCTTCCTTATCAATGAAGCCATCGCGGTTCTGGTCGATCATGTTGAAGGCTTCTTTGAACTCTTGGATCTGGGACTGGTCGAACATGGCAAACACGTTGGACGTGGCCCGCTGGGGGCGCTTTTTGGTGGTTTTTCCCTTAGCCCTTTTGCTAGACATTTTGGCAATTCTTTCCTATTGAAACTATTCCGGTAAATAAAAAGGCAACAAAGACCTAAACAACAGCTGCCACGCTTGTTATTAAAGCCC

>Myosin_light_chain_Myl12.2b_(Myl12.2b)

GGGCTTTAATAACAAGCGTGGCAGCTGTTGTTTAGGTCTTTGTTGCCTTTTTATTTACCGGAATAGTTTCAATAGGAAAGAATTGCCAAAATGTCTAGCAAAAGGGCTAAGGGAAAAACCACCAAAAAGCGCCCCCAGCGGGCCACGTCCAACGTGTTTGCCATGTTCGACCAGTCCCAGATCCAAGAGTTCAAAGAAGCCTTCAACATGATCGACCAGAACCGCGATGGCTTCATTGATAAGGAAGATCTGCATGACATGCTGGCCTCATTAGGTAAGAACCCCACAGACGAATACCTGGAGGCAATGATGAATGAAGCCCCTGGTCCCATTAACTTCACCATGTTCCTCACAATGTTTGGAGAGAAACTTAATGGCACAGATCCTGAAGATGTTATCAAAAATGCATTTGCTTGTTTTGATGAGGAGGGGACGGGTTTTATTCAGGAGGACTACCTAAGGGAGCTGTTGACCACTATGGGAGACAGGTTCACAGATGAAGAAGTAGACGAGTTGTTCAGAGAGGCCCCCATTGACAAGAAAGGGAACTTCAACTACGTAGAATTCACTCGCATCCTGAAACATGGTGCTAAGGATAAGGATGATTAGGACAAGGCCCAGCACTACTGAAACTAATGAAAACTCTACTATAACACTACAACATATTTAACTTTCTTCTTTTCCCCACATTCTTGGGATCGTGCAAAGGGATACATTAATCAGCAAGGCAAAGTCATTTGACTGTACTCTTAAACAGTCTATGTAGGCTTGTACATAACTATGAAATATATAGACATGTAATCTGTGGTAAACCTCAAAGTGAATGAATGCTGTTCTGGTATGTAAATGTGGGAAATAAATTCTGGAATTATT

>Myosin_light_chain_Myl13_(Myl13)

accctgtgctgtctccgtccctctgtgttgcatcgtaaccatcatcatggcccccaagaagaaggaagagcccaaaccagtagctgcacccaagcccccagagcctgagccccccaaggagccagagtttaacccggctgaagtcaagattgaattcaccgctgagcagattgaggatttcaaggatgcgttccaactgtttgacaggacgccgaccaacgagatgaaaatcacctatgctcagtgtggtgatctgatccgggctttgggtcagaaccccaccaacgctgaagttatgcacgtcttaggcaaacccaaagctgaagaaatgcaagtgaagatgctggattttgaacagttcttgcccttgcatcaacacatctgcaaggccaaagaccgcggcacctttgaagacttcgttgagggcctgagagtgtttgacaaagagggcaatgggacagtgatgggtgctgaactcagacacgtcctcgctaccctgggtgagaagatgaaggaagatgaggtcgagcagctgatggctggacaggaggatgccaacggctgtataaactacgaagcttttgtaaaacacatcatggctggctgaa

>Myosin_light_chain_Myl10_(Myl10)

CCAGGAACAGAGGAAAGAGAAGCTCTACACAATGGCACCAAAGAAGGCCAAGAAGAAGGAAGCTGCCAGCTCCAATGTGTTTAGCATGTTTTGAGCAGTCGCAGATCCAGGAGTTCAAAGAGGCTTTCACCATCATGGACCAGAACAGAGATGGCTTCATTGACAAAAACGACCTGAGGGACACATTTGCTGCTTTGGGACGTCTCAACGTTGGCAACGATGAGCTTGATGAAATGCTAAAGGAAGCCTCCGGCCCTATTAACTTCACCGTCTTCCTCACCATGTTTGGAGAGAAGCTCAAAGGTACAGACCCTGAGGAGACTATTCTCAATGCCTTTAAGATCTTCGACCCAGAGGGTACAGGAATCCTTAAAGGAGAAGAGATAAAATATCACCTTATGTCCCAGGCAGACAAATTTACAGAGGCTGAGGTAAACCAGATGTTCACAAACTTTCCCTTGGATGTGGCTGGCAACCTGGATTACAAAAACCTGTGCTACGTCATCACCCACGGAGAAGATAAAGAACAGGAGTAAAAACTGCCTTTCCCATATTTCTCCATGTTTGTTCACGTCCATTTGTGTGTCGTCTTCTCCTTAAGCTCTGTATCCGCCTTTTGTCAATAAAGAGATAAAAAAAC

>Myosin_light_chain_Myl6_(Myl6)

acactcccgccgcaacacacacccggaaaacaagccactacagaatatgtctgacttcagcgaggaccagattcttgattgagtttaacttggagcaaatacacgagtttaaagaagccttcctcctgtttgaccggacgggagatgggaagatcacctacagtcagtgtggggatgtgatgcgtgcactgggccaaaatcctgtcaatgccgaggtgctcaaagtcctgggcaacccaaaggcagaagaaatgaaccataaattgctggactttgaacagttcctgcccatgctccaggccatcgctaagaacaaagaccagggcacgtttgaggactttgtggaggggctcagagtatttgacaaggagggtaacggcacagtcatgggtgctgagctccgccatgttctcaccacactgggagagaagatgaccgaagaggaggtagaaacgcttctagccggacacgaagatgctaacggttgcatcaattacgaagaactcgtccgcatggtcatgagcggttgaagataaagaccacagctcatttgtttgtcttattttctatgtaataatttctttatatgcattttgttttggtttttcctattcattccatcaccttcatttgcttaatggctccagtccttcgctcgtgtttgcactcatttgggtattattattagaagtgcctttatcttctttaagttcccatttgactccccaagaggtccgcctcctgtcctgtggtattcaaatcatatagcgatatgaaaaagatctcttgtgtgccgagtgatctgcaataaaacctctcaataaagcacattagcacagcagtgtgtatgcaatatccagtgtctcctcaggcatctctgctggactgtgctgtagtagaaatgtcgtgtctggccttttcagaataagttccatttatcaaattgagaaattatggatcggaaatgcaataacattttaattaatttggaatatcctcattgtccaataaagtacattttaaac
